# Facile Synthesis and In Vitro Activity of *N*-Substituted 1,2-Benzisothiazol-3(2*H*)-ones against Dengue Virus NS2BNS3 Protease

**DOI:** 10.3390/pathogens10040464

**Published:** 2021-04-12

**Authors:** Farwa Batool, Muhammad Saeed, Hafiza Nosheen Saleem, Luisa Kirschner, Jochen Bodem

**Affiliations:** 1 Department of Chemistry and Chemical Engineering, Syed Babar Ali School of Science and Engineering, Lahore University of Management Sciences, Lahore 54692, Pakistan; 15130009@lums.edu.pk (F.B.); nosheen.saleem@lums.edu.pk (H.N.S.); 2 Institut für Virologie und Immunbiologie, Versbacher Straße 7, 97078 Würzburg, Germany; luisa.kirschner@uni-wuerzburg.de

**Keywords:** dengue virus, direct-acting antivirals, 1,2-benzisothiazolinone, drug discovery, infectivity assays

## Abstract

Several new *N*-substituted 1,2-benzisothiazol-3(2*H*)-ones (BITs) were synthesised through a facile synthetic route for testing their anti-dengue protease inhibition. Contrary to the conventional multistep synthesis, we achieved structurally diverse BITs with excellent yields using a two-step, one-pot reaction strategy. All the synthesised compounds were prescreened for drug-like properties using the online Swiss Absorption, Distribution, Metabolism and Elimination (SwissADME) model, indicating their favourable pharmaceutical properties. Thus, the synthesised BITs were tested for inhibitory activity against the recombinant dengue virus serotype-2 (DENV-2) NS2BNS3 protease. Dose–response experiments and computational docking analyses revealed that several BITs bind to the protease in the vicinity of the catalytic triad with IC_50_ values in the micromolar range. The DENV2 infection assay showed that two BITs, 2-(2-chlorophenyl)benzo[d]isothiazol-3(2*H*)-one and 2-(2,6-dichlorophenyl)benzo[*d*]isothiazol-3(2*H*)-one, could suppress DENV replication and virus infectivity. These results indicate the potential of BITs for developing new anti-dengue therapeutics.

## 1. Introduction

The epidemics of flaviviral diseases have been recognised for more than 200 years. Among them, mosquito-borne disease prevalence is on the rise in Asia, South America, Africa and along the Atlantic Gulf coasts of the United States. Dengue virus (DENV) is transmitted in humans via the bite of the female *Aedes aegypti* mosquito. It has become one of the most emerging and prevalent pathogens [1] in tropical and subtropical regions. The clinical manifestation of DENV infection is dengue fever (DF), which in most cases is characterised by milder and self-limiting symptoms, such as febrile illness, headache, muscular pain and skin rashes [2]. However, a significant number of DENV infected patients are prone to more advanced and fatal diseases, such as dengue shock syndrome (DSS) and dengue haemorrhagic fever (DHF) [3].

For more than a decade, several viral structural and nonstructural (NS) proteins have been exploited as drug targets for developing direct-acting antivirals (DAAs) [4]. Recently, the serine protease of DENV, i.e., a complex of two nonstructural viral proteins labelled NS2BNS3, has become a valuable target for discovering new anti-dengue therapeutics [5]. The DENV NS2BNS3 protease is an essential viral factor for the co- and post-translational processing and modification of the dengue virus polyprotein [6]. Thus, inhibitors of the NS2BNS3 protease can be developed into DAAs for the treatment of DENV infections. The U.S. Food and Drug Administration (FDA) has already approved several protease inhibitors as antiviral drugs for the treatment of HIV and HCV infections; thus, further supporting the notion of exploiting the DENV NS2BNS3 protease as a drug target [7].

Previous efforts to discover DENV NS2BNS3 protease inhibitors have resulted in the identification of several peptides [8,9], peptidomimetics with electrophilic warheads [10,11] and cyclopeptides [12,13], as well as small organic molecules, such as synthetic compounds [14,15,16,17,18] and natural products [19,20,21], as promising leads. However, none of them has reached clinical application due to unfavourable pharmacokinetics and/or pharmacodynamics. Therefore, research to discover new small organic molecules as potent inhibitors of the DENV protease is still an ongoing endeavour.

**Figure 1:**
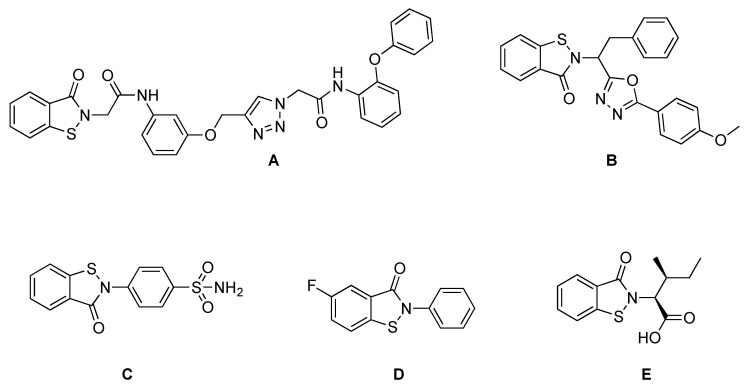
Structures of selected biologically active 1,2-benzisothiazol-3(2*H*)-ones (BITs): triazole-BIT hybrid (**A**), oxadiazole-BIT hybrid (**B**), sulphonamide-BIT hybrid (**C**) (2*S*,3*S*)-3-methyl-2-(3-oxobenzo[d]isothiazol-2(3H)-yl)pentanoic acid (**D**), 5-fluoro-2-phenylbenzo[d]isothiazol-3(2H)-one (**E**).

1,2-Benzisothiazol-3(2*H*)-one (BIT) derivatives are an important class of heterocyclic compounds with a broad range of applications in the field of medicinal chemistry [22,23,24,25]. BIT derivatives (Figure 1) have shown a wide range of biological activities, such as anticancer [26], antimicrobial [27], antifungal [28] and antiviral activities [14,29,30]. Recently, combining two pharmacophores into a single molecule is gaining impetus due to the resulting hybrid’s synergistic effects [16]. For instance, Tiew et al. tethered a BIT scaffold to a triazole ring (Figure 1A) via an *m*-aminophenol linker to yield hybrids, which exhibited inhibitory activities against DENV2 NS2BNS3 at lower micromolar concentrations [14]. Similarly, Lai et al. prepared a series of BIT–oxadiazole hybrids (Figure 1B) and reported their activities as promising inhibitors of DENV and West Nile virus proteases [29]. Encouraged by these findings, we pursued the construction of hybrids of various pharmacophores with a BIT skeleton and planned to accomplish de novo syntheses of the substituted BIT derivatives. Several methods for the synthesis of BITs have already been reported in the literature [31,32,33,34,35,36]; however, these methods involve either multiple steps, are time-consuming or require expensive/toxic reagents. Here, we describe an expeditious synthesis of BIT from thiosalicylic acid (**1**) in a one-pot, two-step reaction strategy. The BITs synthesised in this report were also screened for inhibitory activity against the DENV2 NS2BNS3 protease. The compounds exhibiting antiviral activities in the low-micromolar range were further analysed using computational modelling to map the BIT–protease interactions at the atomic level.

## 2. Methods

### 2.1. Chemistry

Commercial chemicals and reagents (Sigma-Aldrich, Taufkirchen, Germany) were used without any further purification. Ethyl acetate, hexane and dichloromethane (Daejung, Korea) were used for chromatographic analyses after distillation. Purifications were performed using Merck 9385 silica gel (Darmstadt, Germany), pore size 60 Å (70–230 mesh ASTM). Melting points were measured with a Stuart melting point apparatus (Cole-Parmer, Staffordshire, UK) and reported without correction. IR spectra were recorded with an ATR-IR spectrometer (Bruker, Germany). ^1^H and ^13^C NMR were recorded using a AVANCE III HD 600 MHz spectrometer (Bruker, Kloten, Switzerland). Chemical shifts were measured in parts per million using tetramethylsilane (TMS) (Bradbury, Stockport, UK) as standard. Gas chromatography–mass spectrometry (GC–MS) analyses were performed using a Thermo Scientific TRACE 1300 Gas Chromatograph (ThermoFischer Scientific, Waltham, MA, USA).

### 2.2. General Procedure for the Synthesis of 1,2-Benzisothiazol-2(3H)-ones (***5a***–***5q***)

A mixture of thiosalisylic acid (100 mg, 0.65 mmol), thionyl chloride (471.5 µL, 6.5 mmol, 10 equiv.) and a few drops of DMF was treated at 80 °C for 12 h. After removing the excess thionyl chloride, the crude mixture was treated directly with amines (10 equiv.) dissolved in DCM. The resulting solution/suspension was stirred at room temperature for about 20 h. The reaction mixture was diluted with water and extracted with ethyl acetate. The organic layer was dried with magnesium sulfate and the solvent was removed under vacuum to yield the product.

2-Propylbenzo[*d*]isothiazol-3(2*H*)-one (**5a**): Yield 93%; ^1^H NMR (600 MHz, CDCl_3_) *δ*: 7.99 (d, *J* = 7.8 Hz, 1 H), 7.55 (t, *J* = 7.2 Hz, 1 H), 7.35 (t, *J* = 7.2 Hz, 1 H), 7.51 (d, *J* = 8.4 Hz, 1 H), 3.82 (t, *J* = 7.2 Hz, 2 H), 1.75 (m, 1 H), 0.95 (t, *J* = 7.2 Hz, 3 H); ^13^C NMR (150 MHz, CDCl_3_) *δ*: 165.37 (C=O), 140.14 (C), 131.63 (CH), 126.65 (CH), 125.41 (CH), 124.82 (C), 120.30 (CH), 45.53 (CH_2_), 22.90 (CH_2_), 11.12 (CH_3_); EI–MS *m/z* (%): 193 [100, (M^+^)], 151 (100), 137 (16).

2-Butylbenzo[*d*]isothiazol-3(2*H*)-one (**5b**): Yield 89%; ^1^H NMR (600 MHz, CDCl_3_) *δ*: 7.99 (d, *J* = 7.8 Hz, 1 H), 7.55 (t, *J* = 7.8 Hz, 1 H), 7.35 (t, *J* = 7.2 Hz, 1 H), 7.51 (d, *J* = 7.8 Hz, 1 H), 3.82 (t, *J* = 6.6 Hz, 2 H), 1.75 (m, 2 H), 1.34 (m, 2 H), 0.89 (t, *J* = 7.2 Hz, 3 H); ^13^C NMR (150 MHz, CDCl_3_) *δ*: 165.32 (C=O), 140.14 (C), 131.61 (CH), 126.64 (CH), 125.41 (CH), 124.86 (C), 120.29 (CH), 43.67 (CH_2_), 31.58 (CH_2_), 19.82 (CH_2_), 13.65 (CH_3_); EI–MS *m/z*: 207 [37, (M^+^)], 151 (100), 137 (49).

2-Hexylbenzo[*d*]isothiazol-3(2*H*)-one (**5c**): Yield 87%; ^1^H NMR (600 MHz, CDCl_3_) *δ*: 8.00 (d, *J* = 7.8 Hz, 1 H), 7.57 (t, *J* = 7.2 Hz, 1 H), 7.36 (t, *J* = 6.6 Hz, 1 H), 7.51 (d, *J* = 7.8 Hz, 1 H), 3.85 (t, *J* = 7.2 Hz, 2 H), 1.72 (m, 2 H), 1.35 (m, 2 H), 1.25 (m, 4 H), 0.85 (t, *J* = 3.6 Hz, 3 H); ^13^C NMR (150 MHz, CDCl_3_) *δ*: 165.30 (C=O), 140.14 (C), 131.61 (CH), 126.64 (CH), 125.40 (CH), 124.87 (C), 120.29 (CH), 43.98 (CH_2_), 31.37 (CH_2_), 29.51 (CH_2_), 26.26 (CH_2_), 22.48 (CH_2_), 14.0 (CH_3_); EI–MS *m/z*: 235 [100, (M^+^)], 151 (88), 137 (29).

2-Dodecylbenzo[*d*]isothiazol-3(2*H*)-one (**5d**): Yield 94%; ^1^H NMR (600 MHz, CDCl_3_) *δ*: 8.01 (d, *J* = 7.8 Hz, 1 H), 7.56 (m, 1 H), 7.36 (t, *J* = 7.2, 1 H), 7.51 (d, *J* = 8.4, 1 H), 3.86 (t, *J* = 7.2 Hz, 1 H), 1.73 (t, *J* = 7.2, 2 H), 1.21–1.34 (m, 18 H), 0.84 (t, *J* = 6.6, 3 H); ^13^C NMR (150 MHz, CDCl_3_) *δ*: 165.34 (C=O), 140.15 (C), 131.64 (CH), 126.67 (CH), 125.43 (CH), 124.84 (C), 120.28 (CH), 44.04 (CH_2_), 31.91 (CH_2_), 30.93 (CH_2_), 29.71 (CH_2_), 29.62 (CH_2_), 29.55 (CH_2_), 29.46 (CH_2_), 29.34 (CH_2_), 29.20 (CH_2_), 26.60 (CH_2_), 22.69 (CH_2_), 14.12 (CH_3_); EI–MS *m/z*: 319 [30, (M^+^)], 151 (100), 136 (57).

2-(2-Hydroxyethyl)benzo[*d*]isothiazol-3(2*H*)-one (**5e**): Yield 86%, mp 110–112 °C; ^1^H NMR (600 MHz, CDCl_3_) *δ*: 7.99 (d, *J* = 8.4 Hz, 1 H), 7.58 (m, 1 H), 7.37 (m, 1 H), 7.51 (d, *J* = 8.4, 1 H), 3.93 (t, *J* = 5.4 Hz, 2 H), 4.02 (t, *J* = 4.8 Hz, 2 H), 2.67 (s, 1 H); ^13^C NMR (150 MHz, CDCl_3_) *δ*: 163.38 (C=O), 139.71 (C), 130.95 (CH), 125.60 (CH), 124.57 (CH), 123.18 (C), 119.21 (CH), 60.91 (CH_2_), 46.44 (CH_2_); EI–MS *m/z*: 195 [88, (M^+^)], 163 (91), 151 (83), 137 (71), 136 (100).

2-Benzylbenzo[*d*]isothiazol-3(2*H*)-one (**5f**): Yield 77% mp 87–89 °C; ^1^H NMR (600 MHz, CDCl_3_) *δ*: 8.05 (d, *J* = 7.8 Hz, 1 H), 7.56 (t, *J* = 7.8 Hz, 1 H), 7.38 (t, *J* = 7.2 Hz, 1 H), 7.46 (d, *J* = 7.8 Hz, 1 H), 5.04 (s, 2 H), 7.34–7.29 (m, 5 H); ^13^C NMR (150 MHz, CDCl_3_) *δ*: 165.35 (C=O), 140.42 (C), 136.19 (C), 131.87 (CH), 128.86 (CH), 128.46 (CH), 128.31 (CH), 126.84 (CH), 125.53 (CH), 124.50 (C), 120.42 (CH), 47.59 (CH_2_); EI–MS *m/z*: 241 [100, (M^+^)], 136 (79), 91 (88).

2-(4-Butylphenyl)benzo[*d*]isothiazol-3(2*H*)-one (**5g**): Yield 92%, mp 168–172 °C; ^1^H NMR (600MHz, CDCl_3_) *δ*: 7.76 (d, *J* = 8.4 Hz, 1 H), 7.31 (t, *J* = 7.2 Hz, 1 H), 7.55 (m, 1 H), 7.45 (d, *J* = 7.2 Hz, 1 H), 7.40 (d, *J* = 7.2 Hz, 2 H), 7.13 (d, *J* = 8.4 Hz, 2 H), 2.57 (t, *J* = 7.8 Hz, 2 H), 1.57 (m, 2 H), 1.33 (m, 2 H), 0.91 (t, *J* = 7.2 Hz, 3 H); ^13^C NMR (150 MHz, CDCl_3_) *δ*: 163.23 (C=O), 141.28 (C), 133.53 (C), 131.22 (C), 128.29 (CH), 127.86 (CH), 126.12 (CH), 124.75 (CH), 123.76 (CH), 119.26 (C), 119.03 (CH), 32.46 (CH_2_), 28.68 (CH_2_), 21.29 (CH_2_), 12.92 (CH_3_); EI–MS (EI) *m/z*: 283 [44, (M^+^)], 282 (77), 240 (100).

2-(2-Nitrophenyl)benzo[*d*]isothiazol-3(2*H*)-one (**5h**): Yield 78%, mp 199–200 °C; ^1^H NMR (600 MHz, CDCl_3_) *δ*: 8.07 (d, *J* = 4.8 Hz, 2 H), 7.71 (m, 2 H), 7.63 (m, 1 H), 7.55 (m, 1 H), 7.53 (m, 1 H), 7.44 (t, *J* = 7.2, 1 H); ^13^C NMR (150 MHz, CDCl_3_) *δ*: 164.65 (C=O), 146.84 (C), 141.02 (C), 133.86 (CH), 132.91 (CH), 130.03 (CH), 129.71 (C), 129.33 (CH), 127.61 (CH), 126.13 (CH), 125.84 (CH), 122.97 (C), 120.35 (CH); EI–MS *m/z*: 272 [35, (M^+^)], 152 (100).

2-(4-Nitrophenyl)benzo[d]isothiazol-3(2*H*)-one (**5i**): Yield 82%, mp 126–129 °C; ^1^H NMR (600 MHz, CDCl_3_) *δ*: 8.09 (d, *J* = 7.8 Hz, 1 H), 7.70 (m, 1 H), 7.46 (m, 1 H), 7.59 (d, *J* = 7.8 Hz, 1 H), 8.02 (m, 2 H), 8.31 (m, 2 H); ^13^C NMR (150 MHz, CDCl_3_) *δ*: 164.32 (C=O), 133.36 (CH), 127.58 (CH), 126.41 (CH), 124.97 (CH), 124.51 (C), 122.90 (CH), 120.19 (CH); EI–MS *m/z*: 272 [100, (M^+^)], 271 (36), 242 (31).

2-(2-Methyl-4-nitrophenyl)benzo[*d*]isothiazol-3(2*H*)-one (**5j**): Yield 81%, mp 135–138 °C; ^1^H NMR (600 MHz, CDCl_3_) *δ*: 7.05 (d, *J* = 7.8 Hz, 1 H), 7.70 (t, *J* = 7.2, 1 H), 7.56 (d, *J* = 7.8, 1 H), 7.48 (t, *J* = 7.2, 1 H), 7.46 (d, *J* = 8.4, 1 H), 8.10 (d, *J* = 8.4, 1 H), 8.23 (s, 1 H), 2.35 (s, 3 H); ^13^C NMR (150 MHz, CDCl_3_) *δ*: 164.56 (C=O), 146.71 (C), 145.99 (C), 141.12 (C), 135.80 (C), 132.81 (CH), 132.08 (CH), 127.44 (CH), 126.20 (CH), 124.72 (CH), 124.17 (CH), 123.15 (CH), 120.46 (C), 18.62 (CH_3_); EI–MS *m/z*: 286 [78, (M^+^)], 270 (29), 269 (100).

2-(4-Bromophenyl)benzo[*d*]isothiazol-3(2*H*) (**5k**): Yield 84%, mp 138–140 °C; ^1^H NMR (600 MHz, CDCl_3_) *δ*: 8.07 (d, *J* = 7.8 Hz, 1 H), 7.65 (t, *J* = 7.8, 1 H), 7.43 (t, *J* = 7.8, 1 H), 7.58 (m, 5 H); ^13^C NMR (150 MHz, CDCl_3_) *δ*: 163.07 (C=O), 138.58 (C), 135.37 (C), 131.59 (CH), 131.42 (CH), 126.26 (CH), 124.98 (CH), 124.82 (CH), 123.61 (C), 119.31 (C), 119.11 (CH); EI–MS *m/z* (%): 305 [62, (M^+^)], 307 (100), 306 (46), 304 (72), 226 (82).

2-(2-Hydroxyphenyl)benzo[*d*]isothiazol-3(2*H*)-one (**5l**): Yield 77%, mp 198–200 °C; ^1^H NMR (600 MHz, CDCl_3_) *δ*: 8.11 (d, *J* = 7.8 Hz, 1 H), 7.48 (m, 1 H), 7.69 (m, 1 H), 7.61 (d, *J* = 7.2, 1 H), 7.36 (dd, *J* = 7.9, 1 H), 7.24 (m, 1 H), 6.95 (m, 1 H), 7.01 (dd, *J* = 8.2, 1 H); ^13^C NMR (150 MHz, CDCl_3_) *δ*: 165.03 (C=O), 151.69 (C), 141.77 (C), 132.70 (CH), 129.56 (CH), 127.20 (CH), 126.23 (CH), 125.37 (CH), 123.89 (CH), 121.39 (C), 121.33 (CH), 120.09 (CH); EI–MS *m/z*: 243 [95, (M^+^)], 136 (100).

2-(2-Chlorophenyl)benzo[d]isothiazol-3(2*H*)-one (**5m**): Yield 68%, mp 117–119 °C; ^1^H NMR (600 MHz, CDCl_3_) *δ*: 8.10 (d, *J* = 7.8 Hz, 1 H), 7.66 (m, 1 H), 7.38 (m, 1 H), 7.57 (d, *J* = 7.8 Hz, 1 H), 7.53 (dd, *J* = 5.4Hz, 1 H), 7.39 (m, 2 H), 7.42 (dd, *J* = 6.0Hz, 1 H); ^13^C NMR (150 MHz, CDCl_3_) *δ*: 163.80 (C=O), 140.36 (C), 133.08 (C), 132.66 (C), 131.45 (CH), 130.07 (CH), 129.72 (CH), 129.68 (CH), 126.80 (CH), 126.38 (CH), 124.73 (CH), 122.32 (C), 119.27 (CH); EI–MS *m/z*: 261 [69, (M^+^)], 226 (100).

2-(2,6-Dichlorophenyl)benzo[*d*]isothiazol-3(2*H*)-one (**5n**): Yield 92%, mp 188–190 °C; ^1^H NMR (600 MHz, CDCl_3_) *δ*: 8.12 (d, *J* = 7.8 Hz, 1 H), 7.68 (m, 1-H), 7.35 (t, *J* = 7.8 Hz, 1H), 7.59 (d, *J* = 7.8 Hz, 1 H), 7.45 (m, 3 H); ^13^C NMR (150 MHz, CDCl_3_) *δ*: 163.53 (C=O), 140.87 (C), 135.75 (CH), 131.65 (CH), 130.55 (C), 130.21 (CH), 127.92 (CH), 126.58 (CH), 124.67 (CH), 122.0 (C), 119.53 (CH); EI–MS *m/z*: 295 [53, (M^+^)], 260 (100).

2-(4-Methoxyphenyl)benzo[d]isothiazol-3(2*H*)-one (**5o**): Yield 89%, mp 146–148 °C; ^1^H NMR (600 MHz, CDCl_3_) *δ*: 8.07 (d, *J* = 7.8 Hz, 1 H), 7.63 (m, 1 H), 7.53 (m, 3 H), 7.41 (t, *J* = 7.2 Hz, 1 H), 6.96 (d, *J* = 9.0 Hz, 2 H), 3.82 (s, 3 H); ^13^C NMR (150 MHz, CDCl_3_) *δ*: 164.31 (C=O), 158.74 (C), 140.06 (C), 132.16 (CH), 129.69 (C), 127.14 (CH), 126.84 (CH), 125.74 (CH), 124.66 (C), 120.08 (CH), 114.61 (CH), 55.57 (CH_3_); EI–MS *m/z*: 257 [83, (M^+^)], 257 (83), 242 (100).

### 2.3. Computational Assessment of the Absorption, Distribution, Metabolism and Elimination (ADME) Properties of BITs

Please see the supporting information for the computational measurements of the ADME parameters. 

### 2.4. Biological Evaluation

#### Inhibition of the DENV2 NS2BNS3 Protease by BITs

The DENV NS2BNS3 activity inhibition assays were conducted in a 96-well plate containing 100 μL total reaction mixture. The reaction was conducted in a buffer containing 50 mM Tris, pH 9.0, 10 mM NaCl, 25% glycerol, 50 μM Bz-Nle-Lys-Arg-Arg-AMC and 1 mM CHAPS in the presence of BITs (200 μg/mL). The reaction, in triplicates, was initiated by the addition of 100 nM DENV2 NS2B-NS3, and the release of fluorogenic AMC was measured at 60 s intervals using a spectrofluorometer for a maximum reaction time of 30 min. A solvent (DMSO) control was run for each assay plate by measuring the activity of DENV NS2BNS3 in the absence of BITs (DMSO control). The effect of background fluorescence was measured by preparing a calibration curve, which in turn was obtained by spiking known concentrations of free AMC in the same volume of the reaction mixtures containing the buffer, enzyme and compounds, and comparing the slope of this curve with that obtained using free AMC concentrations in reaction mixtures containing the buffer, enzyme, no compound and no substrate. The percentage of inhibition was calculated using the measurement of the initial rates of reaction in the presence $\left(v_{\exp}\right)$, or absence $\left(v_{\mathrm{pro}}\right)$ of BIT according to the following formula:

$$\mathrm{Inhibition}\;\left(\%\right)=\;100-\left(\frac{v_{\exp}}{v_{\mathrm{pro}}}\times 100\right).$$

The significance of the observed inhibitions was determined using Student’s *t*-test by comparing the negative control with the values obtained with a single compound.

For the dose–response experiments, the assays were conducted as described above in the presence of threefold serial dilutions of BITs, ranging from 0.076–500 μM concentrations in DMSO. The final concentration of DMSO in each reaction mixture was less than 5%. IC_50_ values were obtained by plotting the residual activity of the DENV protease vs. log of the concentration of BITs by using GraphPad Prism v.7 software (San Diego, CA USA). For the positive control, aprotinin was used in a 5 μM concentration. All experiments were conducted in triplicates. 

### 2.5. Cytotoxicity Measurements Using an MTS Assay

Vero cells were seeded in a 96-well plate (2000 cells/well in 100 μL DMEM) and placed in an incubator maintained at 37 °C in a 5% CO_2_ environment for overnight incubation. The cells were treated with 30 μM (2 µL) of BITs for 2 days at 37 °C in the incubator. Control wells received an equivalent amount of DMSO without the test compound. After two days, each well was directly treated with 50 μL of the CellTiter 96 (Promega, Mannheim, Germany) and incubated for 1–2 h. The absorbance was recorded at 490 nm with a 96-well plate reader (Berthold, Bad Wildbad, Germany).

### 2.6. RNA Isolation and Quantification of DENV

Based on the manufacturer’s manual, viral RNA was isolated from 200 µL cell culture supernatants using the Roche HP Viral Nucleic Acid Kit (Roche, Mannheim, Germany). Relative viral genome copy numbers were determined using 5 µL of the eluted RNA and RT-qPCR using the LightMix^®^ Modular Dengue kit (TIB MOLBIOL, Germany) in combination with the LightCycler RNA Process Control Kit (Roche, Mannheim, Germany). All reactions were performed in triplicates on Roche LightCycler96 or Roche LightCycler480 II qPCR machines (Roche, Mannheim, Germany). The relative RNA concentration was determined using an RNA standard curve and the respective cycler software. The quality of the RT-qPCRs was ensured by following the Minimum Information for Publication of Quantitative Digital PCR Experiments guidelines, i.e., the recursion coefficient of the relative standard curve (*r*^2^) was >0.95, and the triplicate assays with a standard deviation of >0.5 were excluded from the analyses. The resulting genome copy numbers were expressed as genome copies per millilitre of the cell culture supernatants. The statistical significance of the reduction in viral titres was calculated using Student’s *t*-test by comparing the viral load of a single compound with the DMSO control. 

### 2.7. Immunofluorescence Staining of Vero Cells Infected with DENV

The inhibition of viral replication was analysed using immunofluorescence staining. Vero cells were preincubated with selected BITs at 30 μM. The cells were subsequently infected with DENV-2 (multiplicity of infection (MOI) of 0.5). Cellular supernatants were collected after 2 days, centrifuged at 2000 rpm for 5 min to remove the detached cells, and titrated on Vero cells. These cells were fixed with 4% paraformaldehyde 3 days after infection. We performed immunofluorescence staining to visualise the infected cells with monoclonal anti-DENV-2 E antibodies. Bound antibodies were detected with a Cy3-conjugated donkey anti-mouse antibody (Dianova, Hamburg, Germany) (1:500). Stained infected cells were counted using a fluorescence microscope, and the viral titres were calculated. The experiment was performed in triplicates. The statistical significance of the reduction of infected cells was calculated using Student’s *t*-test by comparing numbers of infected cells incubated with a compound with the DMSO control. 

### 2.8. Molecular Docking

Molecular docking studies were performed using AutoDock Vina designed and implemented by Dr.Oleg Trott in the Molecular Graphics Lab at The Scripps Research Institute, San Diego, CA, USA [37]. 2D structures of BITs were drawn in ChemDraw, version 12.0, PerkinElmer Massachusetts, USA and converted into 3D structures using Avogadro, version 1.2.0, Pittsburgh, PA, USA. The optimisation of the geometry and minimisation of the energy was accomplished using the MMFF94 force field in Avogadro and then saved in the pdb format. Coordinates of the reported co-crystal structure of DENV3 NS2NS3 (PDB ID: 3U1I) were downloaded from the protein databank (rcsb.org) on 22 June 2019 and then processed with Autodock Tool, where polar hydrogen atoms and Kollman charges were added. The pdbqt files of both DENV3 BS2BNS3 and BITs were prepared using Autodock Tools and submitted for molecular docking by using a volume grid defined by 54 × 58 × 48 Å along the *x*, *y* and *z* sides, respectively, with the central point defined as center_x = 23.463, center_y = −8.442 and center_z = 17.732. The top models with the most negative docking scores (binding energy in kcal/mol) were selected to investigate the binding interactions using PyMol and Ligplot.

## 3. Results and Discussion

### 3.1. Synthesis of BITs

**Scheme 1:**
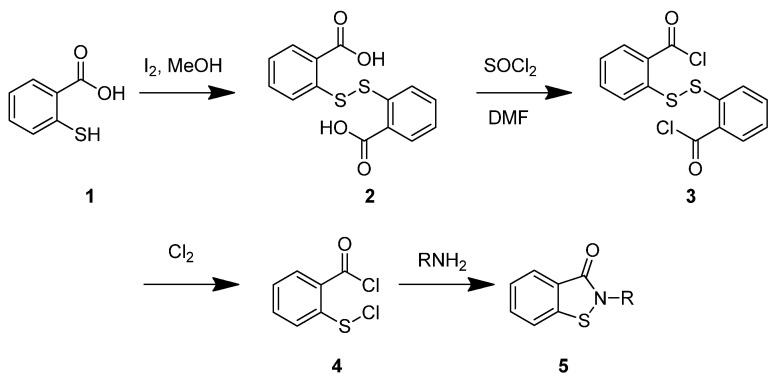
Multistep approach towards the synthesis of BITs.

The synthetic method of Grivas, using thiosalicylic acid (**1**) as the starting material, was the method of choice for the preparation of the BIT derivatives (Scheme 1) [35]. In this multistep approach, **1** was first converted to the disulfide (**2**) with I_2_ in MeOH, and then both the carboxylate groups in **2** were chlorinated with thionyl chloride. Treatment of the generated bisthiosalicylyl chloride (**3**) with elemental halogen (Cl_2_ or Br_2_) in CCl_4_ or sulfuryl chloride resulted in the cleavage of the –S–S– bond to yield 2-chlorosulfenylbenzoyl chloride (**4**). Finally, a double nucleophilic displacement reaction with primary amines afforded the synthesis of the target 1,2-benzisothiazol-3(2*H*)-one (**5**) in four steps total.

**Scheme 2:**
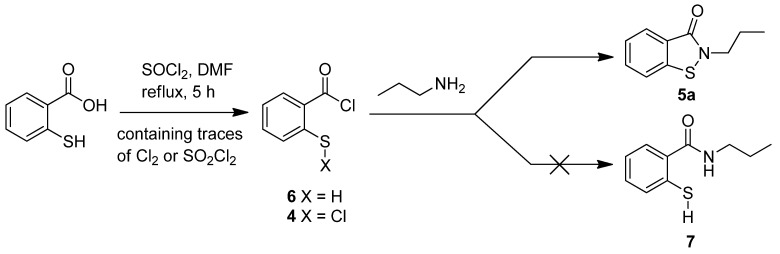
Two-step, one-pot synthesis of BIT **5a** depicting the proposed formation of 2-chlorosulfenyl benzoylchloride (**4**) as an intermediate.

For the synthesis of a library of 2-mercaptobenzamides, we needed 2-mercaptobenzoyl chloride (**6**, X = H) as precursors, which could have been prepared from the reaction of **1** with thionyl chloride [38,39]. Surprisingly, the reaction of *n*-propylamine with the crude reaction mixture, prepared from **1** and thionyl chloride, resulted in the formation of the BIT **5a** as a major (93%) product, rather than the formation of **7** (Scheme 2). A ^1^H NMR investigation showed the absence of both NH and S–H chemical shifts and the EI-MS analysis indicated a molecular ion peak at *m/z* = 193. Additionally, the spectroscopic and physical data (bp = 126–128 °C) matched those previously reported for BIT **5a**. A quick literature search on the predation of **6** (X = H) from **1** using thionyl chloride retrieved only two references [38,39]. In both references, crude **6** was used in the next step without a detailed structural characterisation. Further chemical investigation on this reaction is underway and will be reported elsewhere. Nevertheless, we speculate that the reagent thionyl chloride (SOCl_2_) may contain traces of Cl_2_ or sulfuryl chloride (SO_2_Cl_2_), causing the chlorination of **1** at both the carboxylate and the sulfhydryl (–SH) site to produce 2-chlorosulfenylbenzoyl chloride (**4**), as shown in the Scheme 2. Alternatively, the reaction may have taken place via an entirely different mechanism. Altogether, the reaction of **1** with thionyl chloride produced an intermediate, which reacted with primary amines or anilines, leading to the formation of BITs in excellent yields.

**Scheme 3:**
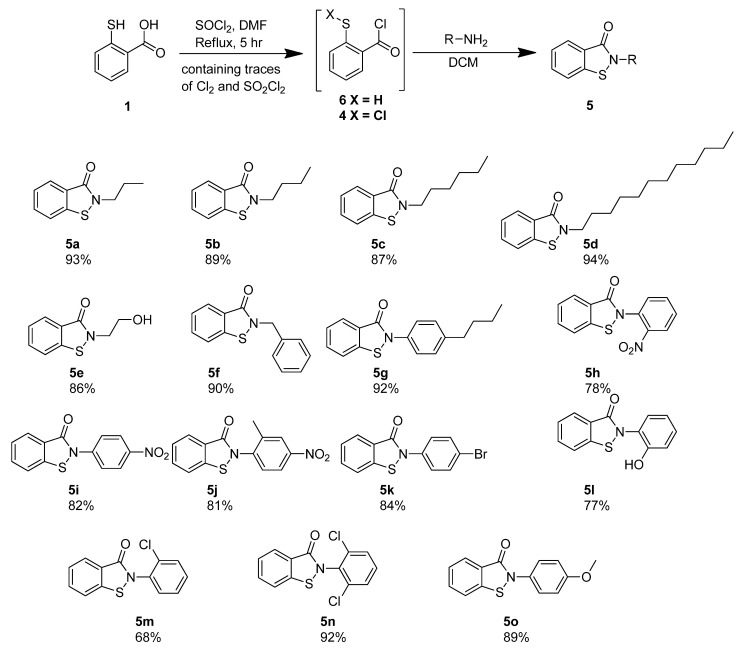
Synthesis of the *N*-substituted BITs with their associated yields.

Following this methodology, we reacted various primary amines and anilines with the crude chlorination mixture and prepared a library of 15 derivatives of 1,2-benzisothiazol-3(2*H*)-ones, as described in Scheme 3.

The structures of all BITs synthesised in this report were elucidated using various spectroscopic techniques. Additionally, the spectral data of the previously reported BITs matched with the ones synthesised via this short route. The ^1^H NMR spectra of compounds **5a**–**o** displayed the characteristic chemical shifts, ranging from *δ* 7.10 to 8.30 ppm for the aromatic ring of 1,2-benzisothiazol-3(2*H*)-one, as well as the chemical shifts of the *N*-substituted R groups (aliphatic and aromatic) in their respective parts per million range. 1,2-Benzisothiazol-3(2*H*)-ones with aliphatic substitutions had their aliphatic region peaks in the range of *δ* 0.8–3.9 ppm, while those with the aromatic substitutions had some of the peaks overlapping the benzene ring peaks of 1,2-benzisothiazol-3(2*H*)-one moiety. The ^13^C spectra of compounds **5a**–**o** displayed the characteristic chemical shifts for carbonyl groups, ranging from *δ* 163 to 165 ppm. The compounds with aliphatic substitutions displayed chemical shifts ranging from *δ* 11 to 47 ppm, and those with aromatic substitutions displayed chemical shifts ranging from *δ* 119 to 151 ppm.

### 3.2. Pharmacokinetics Studies: Predictions of the ADME

The potential of a molecule to exhibit pharmacological or therapeutic properties depends on various physicochemical characteristics of the molecule. Nowadays, computational ADME modelling is used to estimate the lead/drug likeliness of desired molecules and predict whether the molecule should initially be placed in the approved drug molecule section. Lipinski’s rule is considered a criterion for the synthesised molecules’ expectation to be an orally active drug. It consists of 5 tenets: the molecular weight of the compound being <500; the number of hydrogen bond acceptors and donors being <10 and <5, respectively; a lipophilicity threshold <4.15; the partition coefficient *C*log*P* < 5. Violating any of these criteria would render the drug inappropriate for oral intake due to bioavailability problems. 

In the current study, we evaluated several parameters to predict the drug-likeness of the synthesised BITs. All the BITs were estimated for the following in silico physicochemical studies: number of rotatable bonds (nROTB), hydrogen bond acceptor (HBA), hydrogen bond donor (HBD), lipophilicity (iLog*P*) and topological polar surface area (TPSA). The in silico percentage absorption was calculated using the reported formula [(%ABS = 109 – (0.345 × TPSA)]. The percentage absorption of the BITs was found to be in the range of 76–92%. Compounds **5a**–**d**, **5f**, **5g**, **5k, 5m** and **5n** showed the highest absorption, while compounds **5h**–**j** showed the lowest in silico absorption.

All the synthesised BITs were observed to follow the Lipinski rule of five. The molecular weight ranged from 193 to 319 (<500), the HBA ranged from 1 to 3 (≤10), the HBD ranged from 0 to 1 (≤5), iLog*P* (lipophilicity) was found to be in the range of 1.92 to 4.51 (≤5) and MLog*P* was found to be in the range of 1.08 to 4.59 (≤4.5), which suggests that these compounds possess good drug likeliness properties upon administration (Table 1). All the compounds exhibited high gastrointestinal absorption and brain permeability, except **5d**, **5e** and **5h**–**j**. Furthermore, all the compounds, except compound **5d**, showed nROTB in the range 1–5 (<10), suggesting good bioavailability. The TPSA of the synthesised BITs ranged between 50.24 and 96.06 Å^2^ (<140 Å^2^), indicating good intestinal absorption. From these parameters, it was concluded that the synthesised BITs would exhibit good drug-like properties (Table 1).

**Figure 2:**
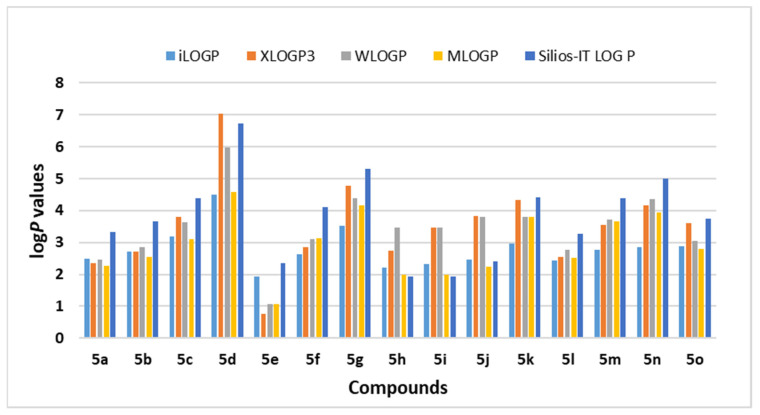
Predicted lipophilicity (log*P* values) of the compounds that were obtained using different calculation methods.

The standard parameter that is used to describe lipophilicity is the partition coefficient between n-octanol and water (log*P*_o/w_) and is considered to be a useful web-based tool for evaluating pharmacokinetics. Multiple computational techniques for log*P*_o/w_ estimation have been developed with varying results on different chemical sets. SwissADME provides access to five free predictive models for lipophilicity, i.e., XLOGP3 (atomic method), WLOGP (atomistic method based upon Wildman and Crippen’s fragmentation method), MLOGP (archetypal topology tool based on a linear relation with 13 molecular descriptors), SILICOS-IT (a hybrid system with 27 fragments and seven topological descriptors) and iLOGP (a physics-based method that uses free solvation energies of n-octanol and water, calculated using the Generalized Born and solvent accessible surface area (GB/SA) model). The log*P*_o/w_ values estimated for each BIT followed similar trends. The values were significantly low in the case of compounds **5e**, **5h** and **5i**. This may be accounted for by the presence of the hydroxyl group and nitro groups in these compounds, which increased the polarity of the compounds. The compound **5d** showed exceptionally high values due to a long aliphatic chain in the molecule. All the remaining compounds showed lipophilicities in a similar range, indicating that the replacement of 4-carbon aliphatic chains with substituted phenyl groups did not significantly alter the lipophilicity. In this study, the log*P*_o/w_ values for the designed compounds were found in the range of +0.77 to +7.04 (Figure 2). These positive values showed that the BITs were highly lipophilic and adhered to the essential criteria for drug-like molecules.

### 3.3. Screening of BITs for Anti-DENV NS2BNS3 Protease Inhibition

The synthesised BITs were tested for their inhibitory activity against the DENV2 NS2B/NS3 protease assay using the previously reported 96-well plate method [16,17,18,19,20]. Briefly, the reaction mixtures (200 μL) containing 50 μM test compound were incubated with 100 nM protease and 5 μM tetrapeptide substrate Bz-Ile-Lys-Arg-Arg-AMC. A reduction in the cleavage of the 4-amino-7-methylcoumarine (AMC) measured in terms of the reduction of fluorescence was deemed to show inhibition of the protease and is reported as a percentage inhibition compared with the solvents’ control reaction. An experiment was conducted to measure the background fluorescence from the organic compounds and the ability of the BITs to quench the AMC fluorescence and lay off the false-positive inhibition (data not shown). No significant background fluorescence and quenching behaviour were observed by any of the tested compounds under the conditions used in these experiments. Furthermore, the enzymatic reactions of the potent inhibitors were repeated by replacing the zwitterionic detergent (CHAPS) with non-ionic (Brij-35) detergent to remove the false-positive results arising due to the possible aggregation of compounds in the experiments. Aprotinine was used as the positive control, as described previously. [18] 

**Figure 3:**
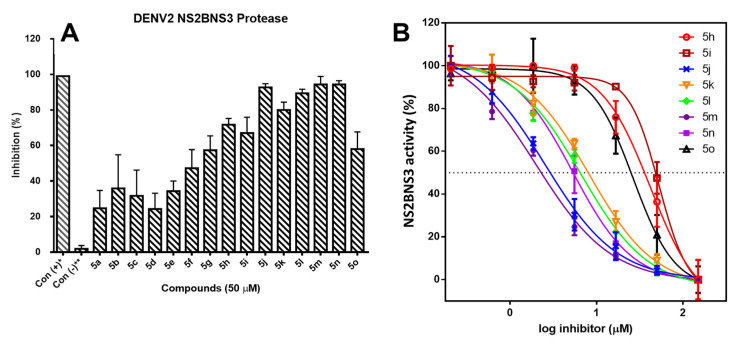
Inhibition and dose–response of DENV NS2BNS3 protease against the BITs. (**A**) A concentration of 50 μM BITs was incubated in the presence of 100 nM recombinant DENV2 NS2BNS3 protease and 5 µM tetrapeptide-AMC substrate in a Tris buffer at pH 9.5. The time-dependent release of fluorescent AMC tag was measured for 30 min and compared with the solvent control (−). The results are displayed as the percentage of inhibition. Aprotinine (5 µM) was used as a positive control. Each bar represents a mean value of three replicates. The observed inhibition with all compounds was significant with *p* < 0.05 for **5a**–**d** and *p* < 0.01 for **5e**–**o** (**B**) Three-fold serial dilutions of the selected BITs were screened between 150 μM and 200 nM concentration in the DENV2 NS2BNS3 protease assay in triplicate. The dashed horizontal line indicates the 50% activity level.

Among all the tested BITs, the ones containing an *N*-substituted aliphatic residue (**5a**–**e**) showed an overall weaker inhibition (<50%) at 50 μM as compared to the ones containing aromatic substitutions (**5f**–**o**) at the N-atom, as shown in Figure 3A. The compounds **5j**, **5m** and **5n** showed the highest percentage (>90%) of inhibition at the tested dose. Although the presence of an electronegative Cl atom (**5m** and **5n**) or O atom (**5l**) at the *ortho-*position of the *N*-substituted benzene ring favoured the inhibition of the DENV-2 protease (≈90% and above), the presence of a NO_2_ group at the same position in **5h** or the *para* position in **5i** showed relatively weaker inhibition (≈70%). Interestingly, the presence of a methyl group at the *ortho* position, albeit with a NO_2_ group athe *para* position in **5i**, enhanced the inhibition of DENV2 NS2BNS3 up to ≈31%, indicating that the weakly electron-donating substituents at the *ortho* position of the *N*-substituted aromatic ring were preferable for enhanced inhibition by BITs. 

The BITs exhibiting more than 65% inhibition at the 50 μM concentration were further investigated for the dose-dependent inhibition of the DENV2 protease. A threefold serial dilution of the selected BITs, ranging from 150 μM to about 200 nM, were screened in triplicate experiments, as described above. The kinetic data were obtained and the protease’s residual activities in the presence of the tested BITs were inserted into the dose–response model rooted in the GraphPad Prism software (version 7.0). The half-maximal inhibitory (IC_50_) dose of the selected compounds was observed within a concentration range of approximately 2–53 μM. Two BITs containing the NO_2_ group at the *ortho* (**5h**) and *para* (**5i**) positions or a methoxy group at the *para* position (**5o**) of the benzene ring demonstrated the highest IC_50_ values of 41.80 ± 1.17 μM, 52.70 ± 3.23 μM and 26.50 ± 4.32 μM, respectively. On the other hand, five BITs (**5j**–**n**) showed lower IC_50_ values ranging between 2 and 8.24 μM (Table 2).

### 3.4. Molecular Docking of Compounds Clustered in the Substrate-Binding Site

The BITs exhibiting promising inhibitory activities in the enzymatic assay were further assessed computationally for an insight into the ligand–receptor binding interactions. For this purpose, the protein sequence of the recombinant DENV-2 protease was submitted for the homology model, which was constructed using the reported structure of DENV-2 protease (PDB ID: 2M9P) as the folding template. Hence, the homology model was folded in the active/closed conformation of the protease and was used for computational modelling using AutoDock Vina.

The structures of **5j**, **5m** and **5n** were drawn in ChemDraw and then imported into Avogadro for conversion into 3D models. The energies were minimised (and the geometries were optimised) by using the MMFF94 force field. The pdbqt files of the protein and ligands were prepared by AutoDock Tools Software and finally submitted for molecular docking using a grid incorporating the whole protein structure.

**Figure 4:**
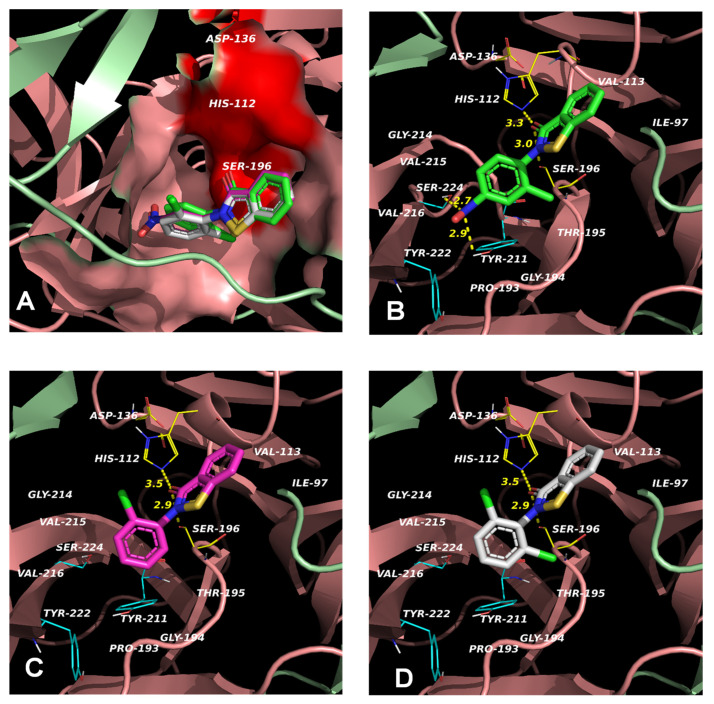
Molecular docking and the predicted mode of binding of BITs with the DENV2 NS2BNS3 protease. The catalytic domain NS3 of the protease is shown in salmon red color, the cofactor NS2B as a pale-green color and the substrate-binding site as surface (**A**) containing the superposed docked poses of **5j**, **5m** and **5n**. The location of the catalytic triad—His112, Asp-136 and Ser196—is depicted in red. (**B**–**D**) Individual docked poses of **5j** (shown in the green tube model), **5m** (magenta) and **5n** (off-white) exhibiting the H-bonding interactions with His-112, Ser-196, Ser-224 and Tyr-211 (shown in yellow). The residues showing the hydrophobic interaction are also marked.

The docked structures were clustered in the substrate-binding site with the binding energies of −7.6, −6.2 and −6.4 kcal/mol for **5j**, **5m** and **5n**, respectively, as shown in Figure 4A. The amide oxygen of compound **5j** was docked in the vicinity of the catalytic triad of the NS3 domain and was found to exhibit H-bonding with His-112 and Ser-196 at distances of 3.3 Å and 3.0 Å, respectively. The aromatic rings of **5j** were found to be surrounded by several nonpolar (e.g., Val-113, Ile-97, Val-215, Val-216 and Pro-193) and aromatic (Tyr-211 and Tyr-222) side chains of the substrate-binding site and contributed towards the favourable hydrophobic and π−π interactions, as shown in Figure 4B. Analogous to the BIT **5j**, the compounds **5m** and **5n** were also docked in the same place with docking scores of −6.2 kcal/mol and −6.4 kcal/mol, respectively. Each of them was also involved in H-bonding with the His-112 and Ser-196 of the catalytic triad at distances of 3.5 Å and 2.9 Å, respectively.

Since **5j**, **5m and 5n** were found to dock at the same location, their benzisothiazolinone ring and the aromatic ring of the *N*-substituents were assumed to be surrounded by identical residues. The difference in the values of the predicted docking scores and the measured IC_50_ values could be assigned to the nature and orientation of substituents on the aromatic rings. For instance, **5j** containing a polar nitro group demonstrated additional interactions with Ser-224 and Tyr-211 residues, thus lowering its binding energy compared to **5m** and **5n**, whereas these interactions were missing in the latter molecules. Nevertheless, the order of the IC_50_ values of these compounds was found to be 2.01 μM for **5m** < 2.56 μM for **5j** < 5.28 μM for **5n,** which is in contradiction with the predicted order of the binding energies as −7.6 kcal/mol for **5j** < −6.4 kcal/mol μM for **5n** < −6.2 kcal/mol for **5m**. This may be accounted for by the presence of a suitably oriented and negatively charged nitro group in compound **5j**, which can enhance favourable binding interactions once at the docking site, but also has to pay a desolvation penalty due to the shedding of the surrounded shell of water molecules in the aqueous environment of the bioassay. This fact was further substantiated in the in vitro studies, where **5j** did not show any activity against DENV2, potentially due to its low uptake by the Vero cells.

### 3.5. In Vitro Testing of the Inhibitory Activity of BITs against the DENV Replication Assay

**Figure 5:**
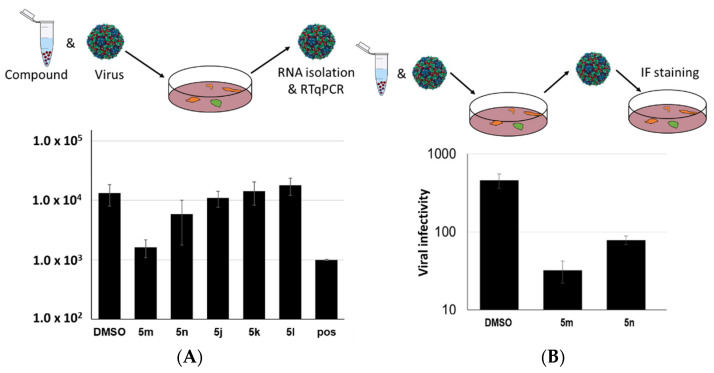
**5m** inhibited DENV replication and viral infectivity. (**A**) Vero cells were infected with DENV2 and incubated in the presence of the compounds. After incubation, viral RNA was isolated and quantified using RT-qPCR. (**B**) Viral infectivity of the compounds **5m** and **5n** was determined using immunofluorescence (IF) staining.

The BITs were tested for cytotoxicity in the Vero cells before their evaluation in cell culture assays (Figure S1). All the BITs showed no cytotoxicity at 30 µM. The BITs showing weaker DENV2 NS2BNS3 protease activity in the biochemical assay were excluded in the in vitro evaluations. To analyse the inhibition of viral replication, Vero cells were pre-incubated for 1 h with the test compounds at a concentration of 30 μM and subsequently infected with DENV2. All infections were performed in triplicate. After two days, the cellular supernatants were collected and the viral RNAs were isolated and quantified using real-time quantitative polymerase chain reaction (RT-qPCR). Among the tested compounds, only **5m** (*p* = 1.1 × 10^−5^) and **5n** (*p* = 0.01) were able to suppress DENV replication at a significant level (Figure 5). 

To further analyse whether the compounds **5m** and **5n** suppressed the viral gene infectivity, Vero cells were pretreated with the compounds and subsequently infected with DENV2 for two days. Cellular supernatants were uses to infect the Vero cells. After two additional days, the infected cells were visualised using immunofluorescence with anti-DENV antibodies. The stained cells were counted (Figure 5). In this assay, **5m** significantly (*p* = 0.003) reduced the viral infectivity by 1.4 orders of magnitude, while the reduction by **5n** was about sixfold (*p* = 0.005). 

Furthermore, to analyse the potential of BITs **5k**–**n** against other flaviviruses, similar experiments were performed with the Zika virus, and none of the tested BITs yielded positive results regarding the Zika virus replication. This observation may lie in the fact that the organisational behaviour is different for both viruses inside of host cells [18,40]. Both DENV and ZIKV viruses have been shown to form dissimilar replication assemblies containing RNA and NS3 such that the ZIKV replication assemblies are additionally surrounded by intermediate filaments in cage-like moieties [20]. This additional support could lead to powerful shielding of the ZIKV protease from inhibitors compared to DENV protease. Therefore, it seemed very likely that the cell-based and in vitro assays’ differences could be traced back to the cellular metabolism and that compounds **5m** and **5n** may serve as lead compounds in flaviviral protease drug discovery.

## 4. Conclusions

In summary, a library of BITs was synthesised using a one-pot, two-step method in a very fast way and tested for their potential against the DENV2 NS2BNS3 protease. The compounds exhibiting high inhibition at 50 µM were investigated for their IC_50_ values, which were found in the range of 2 to 8.25 µM. The BITs demonstrating a lower IC_50_ were further screened by the in vitro viral (DENV and ZIKV) assays, and compound **5m** was found to reduce the viral infectivity by a log value of 1. Computational assessments were also performed, which indicated that these compounds bound at the catalytic site of the NS2BNS3 protease. These preliminary results indicate the potential of BITs for their further development into anti-dengue therapeutics.

## Figures and Tables

**Table 1:** Pharmacokinetics/ADME (absorption, distribution, metabolism and elimination) predictions of the target BITs.

Lipinski Parameters										
Compounds	MW ^a^	HBA ^b^	HBD ^c^	iLog*P* ^d^	Violations	nROTB ^e^	TPSA ^f^	%ABS ^g^	BBB ^h^	GI ABS ^i^
**5a**	193	1	0	2.49	0	2	50.24	91.67	Yes	High
**5b**	207	1	0	2.72	0	3	50.24	91.67	Yes	High
**5c**	235	1	0	3.19	0	5	50.24	91.67	Yes	High
**5d**	319	1	0	4.51	1	11	50.24	91.67	No	High
**5e**	195	2	1	1.92	0	2	70.47	84.69	No	High
**5f**	241	1	0	2.64	0	2	50.24	91.67	Yes	High
**5g**	283	1	0	3.52	1	4	50.24	91.67	Yes	High
**5h**	272	3	0	2.21	0	2	96.06	75.86	No	High
**5i**	272	3	0	2.31	0	2	96.06	75.86	No	High
**5j**	286	3	0	2.45	0	2	96.06	75.86	No	High
**5k**	306	1	0	2.96	0	1	50.24	91.67	Yes	High
**5l**	243	2	1	2.44	0	1	70.47	84.69	Yes	High
**5m**	262	1	0	2.76	0	1	50.24	91.67	Yes	High
**5n**	296	1	0	2.84	0	1	50.24	91.67	Yes	High
**5o**	257	2	0	2.88	0	2	59.47	88.49	Yes	High

^a^ Molecular weight. ^b^ Hydrogen bond acceptor. ^c^ Hydrogen bond donor. ^d^ Partition coefficient. ^e^ Number of rotatable bonds. ^f^ Topological polar surface area. ^g^ Absorption percentage. ^h^ Blood–brain barrier. ^i^ Gastro-intestinal absorption.

**Table 2:** Percent inhibition and IC_50_ values of the selected BITs.

#	Compound	Inhibition ^a^	IC_50_^b^
		(%)	(μM)
1	**5h**	72	41.80 ± 1.17
2	**5i**	67	52.70 ± 3.23
3	**5j**	**92**	**2.56 ± 1.03**
4	**5k**	80	8.24 ± 1.16
5	**5l**	89	6.66 ± 2.17
6	**5m**	**94**	**2.01± 0.98**
7	**5n**	**94**	**5.28 ± 1.89**
8	**5o**	58	26.50 ± 4.32

^a^ Inhibition of the protease at a single dose (50 μM) of BIT. ^b^ IC_50_ values are the concentrations at which the DENV protease is inhibited by 50%. Bold values: the most potent inhibitors of all BITs.

## Data Availability

Not applicable.

## Supplementary Materials

The following are available online at https://www.mdpi.com/article/10.3390/pathogens10040464/s1. Figure S1: MTS viability assays with the compounds. Vero cells were incubated with the compounds at a concentration of 30 µM for 72 h. The MTS substrate was added and the cell were further incubated for 1 h. The absorbance was measured at 490 nm.

## References

[1] Bhatt S., Gething P.W., Brady O.J., Messina J.P., Farlow A.W., Moyes C.L., Drake J.M., Brownstein J.S., Hoen A.G., Sankoh O. (2013). The global distribution and burden of dengue. Nature.

[2] Timiri A.K., Sinha B.N., Jayaprakash V. (2016). Progress and prospects on DENV protease inhibitors. Eur. J. Med. Chem..

[3] Wilder-Smith A., Gubler D.J., Weaver S.C., Monath T.P., Heymann D.L., Scott T.W. (2017). Epidemic arboviral diseases: Priorities for research and public health. Lancet Infect. Dis..

[4] Qadir A., Riaz M., Saeed M., Shahzad-Ul-Hussan S. (2018). Potential targets for therapeutic intervention and structure based vaccine design against Zika virus. Eur. J. Med. Chem..

[5] Nitsche C., Holloway S., Schirmeister T., Klein C.D. (2014). Biochemistry and medicinal chemistry of the dengue virus protease. Chem. Rev..

[6] Yusof R., Clum S., Wetzel M., Murthy H.M., Padmanabhan R. (2000). Purified NS2B/NS3 serine protease of dengue virus type 2 exhibits cofactor NS2B dependence for cleavage of substrates with dibasic amino acids in vitro. J. Biol. Chem..

[7] Liu H., Wu R., Sun Y., Ye Y., Chen J., Luo X., Shen X., Liu H. (2014). Identification of novel thiadiazoloacrylamide analogues as inhibitors of dengue-2 virus NS2B/NS3 protease. Bioorg. Med. Chem..

[8] Da Silva-Junior E.F., de Araujo-Junior J.X. (2019). Peptide derivatives as inhibitors of NS2B-NS3 protease from Dengue, West Nile, and Zika flaviviruses. Bioorg. Med. Chem..

[9] Nitsche C., Behnam M.A., Steuer C., Klein C.D. (2012). Retro peptide-hybrids as selective inhibitors of the Dengue virus NS2B-NS3 protease. Antivir. Res..

[10] Yin Z., Patel S.J., Wang W.L., Wang G., Chan W.L., Rao K.R., Alam J., Jeyaraj D.A., Ngew X., Patel V. (2006). Peptide inhibitors of Dengue virus NS3 protease. Part 1: Warhead. Bioorg. Med. Chem. Lett..

[11] Drazic T., Kopf S., Corridan J., Leuthold M.M., Bertosa B., Klein C.D. (2020). Peptide-beta-lactam Inhibitors of Dengue and West Nile Virus NS2B-NS3 Protease Display Two Distinct Binding Modes. J. Med. Chem..

[12] Takagi Y., Matsui K., Nobori H., Maeda H., Sato A., Kurosu T., Orba Y., Sawa H., Hattori K., Higashino K. (2017). Discovery of novel cyclic peptide inhibitors of dengue virus NS2B-NS3 protease with antiviral activity. Bioorg. Med. Chem. Lett..

[13] Lin K.H., Ali A., Rusere L., Soumana D.I., Kurt Yilmaz N., Schiffer C.A. (2017). Dengue Virus NS2B/NS3 Protease Inhibitors Exploiting the Prime Side. J. Virol..

[14] Tiew K.C., Dou D., Teramoto T., Lai H., Alliston K.R., Lushington G.H., Padmanabhan R., Groutas W.C. (2012). Inhibition of Dengue virus and West Nile virus proteases by click chemistry-derived benz[d]isothiazol-3(2H)-one derivatives. Bioorg. Med. Chem..

[15] Aravapalli S., Lai H., Teramoto T., Alliston K.R., Lushington G.H., Ferguson E.L., Padmanabhan R., Groutas W.C. (2012). Inhibitors of Dengue virus and West Nile virus proteases based on the aminobenzamide scaffold. Bioorg. Med. Chem..

[16] Hamdani S.S., Khan B.A., Hameed S., Batool F., Saleem H.N., Mughal E.U., Saeed M. (2020). Synthesis and evaluation of novel S-benzyl- and S-alkylphthalimide- oxadiazole -benzenesulfonamide hybrids as inhibitors of dengue virus protease. Bioorg. Chem..

[17] Weng Z., Shao X., Graf D., Wang C., Klein C.D., Wang J., Zhou G.C. (2017). Identification of fused bicyclic derivatives of pyrrolidine and imidazolidinone as dengue virus-2 NS2B-NS3 protease inhibitors. Eur. J. Med. Chem..

[18] Wu H., Bock S., Snitko M., Berger T., Weidner T., Holloway S., Kanitz M., Diederich W.E., Steuber H., Walter C. (2015). Novel dengue virus NS2B/NS3 protease inhibitors. Antimicrob. Agents Chemother..

[19] Bharadwaj S., Lee K.E., Dwivedi V.D., Yadava U., Panwar A., Lucas S.J., Pandey A., Kang S.G. (2019). Discovery of Ganoderma lucidum triterpenoids as potential inhibitors against Dengue virus NS2B-NS3 protease. Sci. Rep..

[20] Saleem H.N., Batool F., Mansoor H.J., Shahzad-ul-Hussan S., Saeed M. (2019). Inhibition of Dengue Virus Protease by Eugeniin, Isobiflorin, and Biflorin Isolated from the Flower Buds of Syzygium aromaticum (Cloves). ACS Omega.

[21] Sarwar M.W., Riaz A., Dilshad S.M.R., Al-Qahtani A., Nawaz-Ul-Rehman M.S., Mubin M. (2018). Structure activity relationship (SAR) and quantitative structure activity relationship (QSAR) studies showed plant flavonoids as potential inhibitors of dengue NS2B-NS3 protease. BMC Struct. Biol..

[22] Mor M., Zani F., Mazza P., Silva C., Bordi F., Morini G., Plazzi P.V. (1996). Biological studies on 1,2-benzisothiazole derivatives V. Antimicrobial properties of N-alkanoic, N-arylalkanoic and N-aryloxyalkanoic derivatives of 1,2-benzisothiazolin-3-one: QSAR study and genotoxicity evaluation. Farmaco.

[23] Wright S.W., Petraitis J.J., Abelman M.M., Bostrom L.L., Corbett R.L., Green A.M., Kindt R.M., Sherk S.R., Magolda R.L. (1993). Inhibition of cartilage breakdown by isothiazolones. Bioorg. Med. Chem. Lett..

[24] Matsunaga H., Matsui T., Ohya K., Okino K., Hayashida K., Maebayashi K., Kiriike N., Stein D.J. (2006). A benzisothiazole derivative and antipsychotic agent, perospirone, for augmentation of selective serotonin reuptake inhibitors (SSRIs) in refractory obsessive-compulsive disorder (OCD): Two patient case series. Int. J. Psychiatry Clin. Pract..

[25] Nandakumar N., Gopinath P., Gopas J., Muraleedharan K.M. (2020). Benzisothiazolone Derivatives Exhibit Cytotoxicity in Hodgkin’s Lymphoma Cells through NF-kappaB Inhibition and are Synergistic with Doxorubicin and Etoposide. Anticancer Agents Med. Chem..

[26] Wang L.H., Yang X.Y., Zhang X., Mihalic K., Fan Y.X., Xiao W., Howard O.M., Appella E., Maynard A.T., Farrar W.L. (2004). Suppression of breast cancer by chemical modulation of vulnerable zinc fingers in estrogen receptor. Nat. Med..

[27] Lu J., Vodnala S.K., Gustavsson A.L., Gustafsson T.N., Sjoberg B., Johansson H.A., Kumar S., Tjernberg A., Engman L., Rottenberg M.E. (2013). Ebsulfur is a benzisothiazolone cytocidal inhibitor targeting the trypanothione reductase of Trypanosoma brucei. J. Biol. Chem..

[28] Dou D., Alex D., Du B., Tiew K.C., Aravapalli S., Mandadapu S.R., Calderone R., Groutas W.C. (2011). Antifungal activity of a series of 1,2-benzisothiazol-3(2H)-one derivatives. Bioorg. Med. Chem..

[29] Lai H., Dou D., Aravapalli S., Teramoto T., Lushington G.H., Mwania T.M., Alliston K.R., Eichhorn D.M., Padmanabhan R., Groutas W.C. (2013). Design, synthesis and characterisation of novel 1,2-benzisothiazol-3(2H)-one and 1,3,4-oxadiazole hybrid derivatives: Potent inhibitors of Dengue and West Nile virus NS2B/NS3 proteases. Bioorg. Med. Chem..

[30] Sharmeen L., McQuade T., Heldsinger A., Gogliotti R., Domagala J., Gracheck S. (2001). Inhibition of the early phase of HIV replication by an isothiazolone, PD 161374. Antivir. Res..

[31] Pietka-Ottlik M., Potaczek P., Piasecki E., Mlochowski J. (2010). Crucial role of selenium in the virucidal activity of benzisoselenazol-3(2H)-ones and related diselenides. Molecules.

[32] Correa A., Tellitu I., Dominguez E., SanMartin R. (2006). Novel alternative for the N-S bond formation and its application to the synthesis of benzisothiazol-3-ones. Org. Lett..

[33] Guo J.R., Gong J.F., Song M.P. (2019). Nickel(ii)-catalysed C(sp(2))-H sulfuration/annulation with elemental sulfur: Selective access to benzoisothiazolones. Org. Biomol. Chem..

[34] Siegemund A., Taubert K., Schulze B. (2002). 1,2-Benzisothiazol-3(2H)-ones and heterocyclic annelated isothiazol-3(2H)-ones, Part 11: Synthesis, reaction and biological activity. Sulfur Rep..

[35] Grivas J. (1975). Novel general synthesis of 2-substituted 1, 2-benzisothiazolin-3-ones. Cyclisation of N-substituted 2-methoxycarbonylbenzenesulfenamides. J. Org. Chem..

[36] El-Sabbagh O.I. (2013). Synthesis of some new benzisothiazolone and benzenesulfonamide derivatives of biological interest starting from saccharin sodium. Arch. Der. Pharm..

[37] Trott O., Olson A.J. (2010). AutoDock Vina: Improving the speed and accuracy of docking with a new scoring function, efficient optimisation, and multithreading. J. Comput. Chem..

[38] Singh G., Rani S., Arora A., Aulakh D., Wriedt M. (2016). Thioester-appended organosilatranes: Synthetic investigations and application in the modification of magnetic silica surfaces. New J. Chem..

[39] Wang S., Fang K., Dong G., Chen S., Liu N., Miao Z., Yao J., Li J., Zhang W., Sheng C. (2015). Scaffold Diversity Inspired by the Natural Product Evodiamine: Discovery of Highly Potent and Multitargeting Antitumor Agents. J. Med. Chem..

[40] Millies B., von Hammerstein F., Gellert A., Hammerschmidt S., Barthels F., Goppel U., Immerheiser M., Elgner F., Jung N., Basic M. (2019). Proline-Based Allosteric Inhibitors of Zika and Dengue Virus NS2B/NS3 Proteases. J. Med. Chem..

