# Supplementary material for: Facile Synthesis and In Vitro Activity of N-Substituted 1,2-Benzisothiazol-3(2H)-ones against Dengue Virus NS2BNS3 Protease
Source: Pathogens. 2021 Apr 12;10(4):464. doi: 10.3390/pathogens10040464 (PMC8070447; doi:10.3390/pathogens10040464)
Supplement: Supplementary file 1 [file pathogens-10-00464-s001.pdf]

Facile synthesis and *in vitro* activity of N-substituted 1,2-benzisothiazol-3(2*H*)-ones against Dengue virus NS2BNS3 protease

Farwa Batool,[a] Muhammad Saeed[*,a] Hafiza Nosheen Saleem,[a] Luisa Krishner,[b] and Jochen Bodem[b,*]

[a]Department of Chemistry and Chemical Engineering, Syed Babar Ali School of Science and Engineering, Lahore University of Management Sciences, Lahore, 54692, Pakistan

[b]Institut für Virologie und Immunbiologie, Versbacher Straße 7, 97078 Würzburg, Germany

* To whom correspondence should be addressed. E-mail: muhammad.saeed@lums.edu.pk; jochen.bodem@uni-wuerzburg.de

Supporting information

Table of contents:

- Toxicity data
- ^{1}H & ^{13}C NMR Spectra of compounds **4** and **6**
- ^{1}H & ^{13}C NMR Spectra of 1,2-benzisothiazol-3(2*H*)-ones

Viability test for the compounds on Vero cells

To exclude any toxic side effects of the used compounds, cell survival and metabolism were measured by MTS Cell Viability Assay (Promega). Vero cells ($2x10^4$) were incubated with 30µM of the compounds solubilised in DMEM or DMSO. Wells with either DMEM alone or DMSO served as controls. The MTS Cell Viability Assay was performed according to the manufacturer's instructions in triplicates. After 72h, the substrate was added and cells were incubated for one additional hour. Then the absorbance was measured at 490 nm. Changes in the absorbance were compared to the solvent control (Figure S1). All compounds showed no cytotoxicity at 30µM.

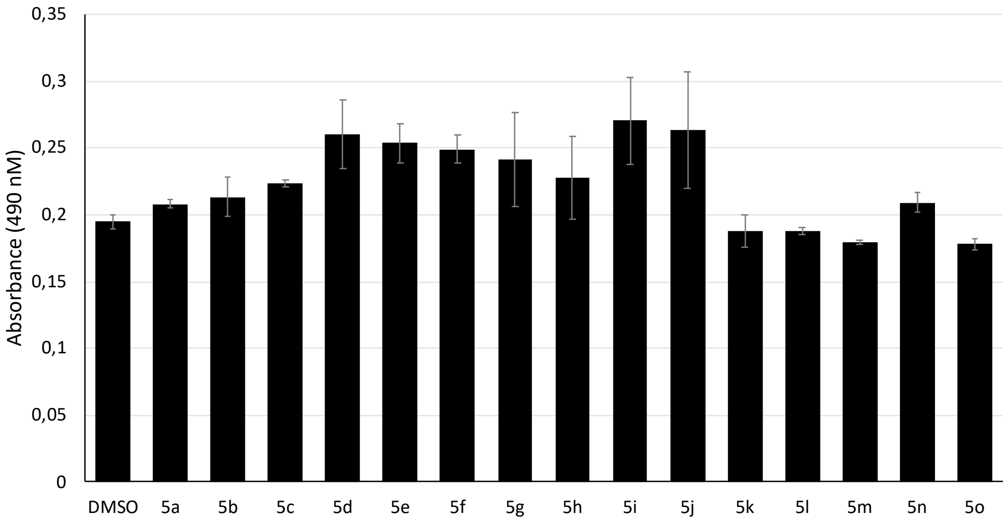

Figure S1: MTS viability assays with the compounds. Vero cells were incubated with the compounds at a concentration of 30µM for 72h. The MTS substrate was added and the cell were further incubated for 1 h. The absorbance was measured at 490nm.

GC-MS of compounds **4** and **6**

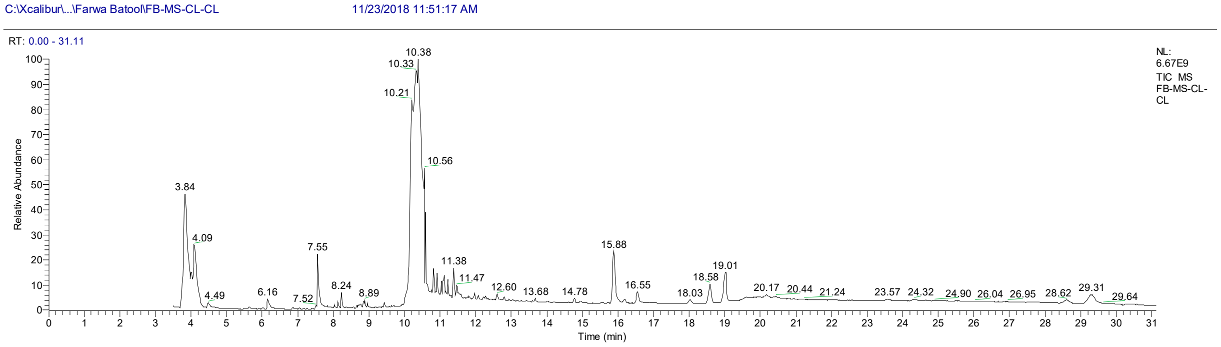

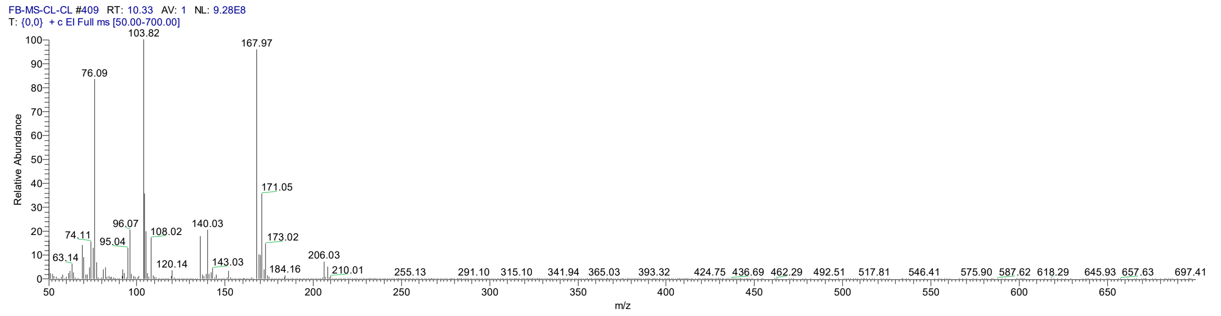

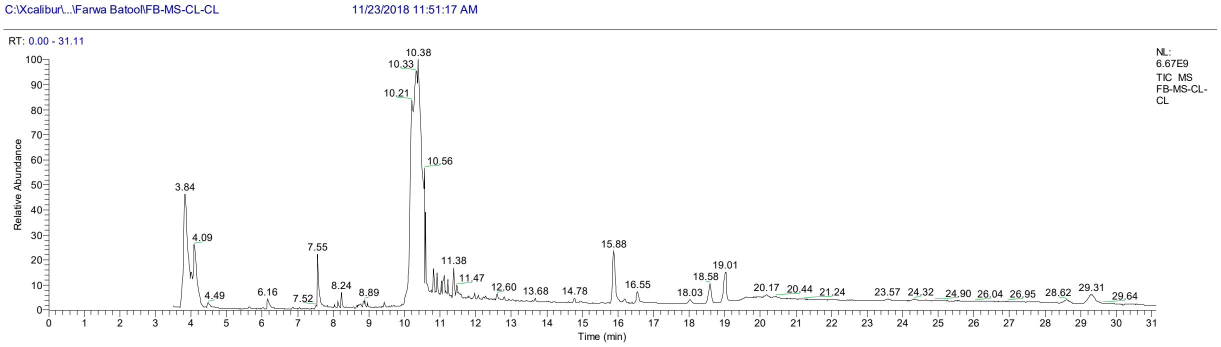

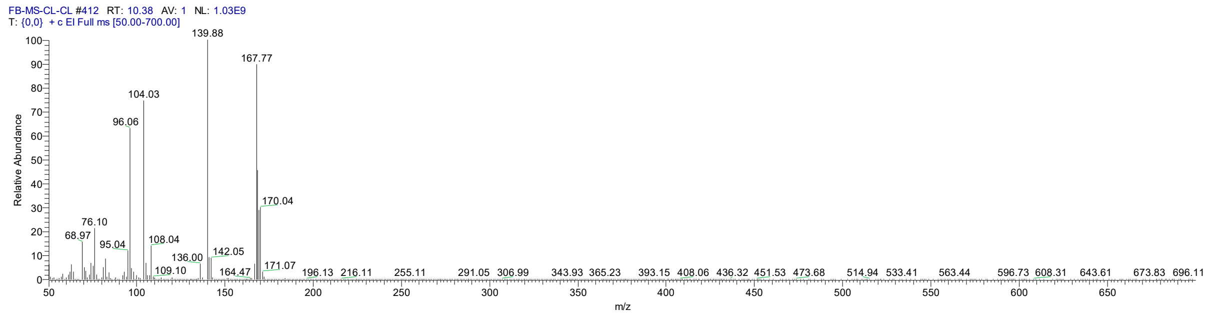

^{1}H & ^{13}C NMR Spectra of compounds **4** and **6**

2-(Chlorosulfenyl)benzoyl chloride (4)

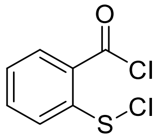

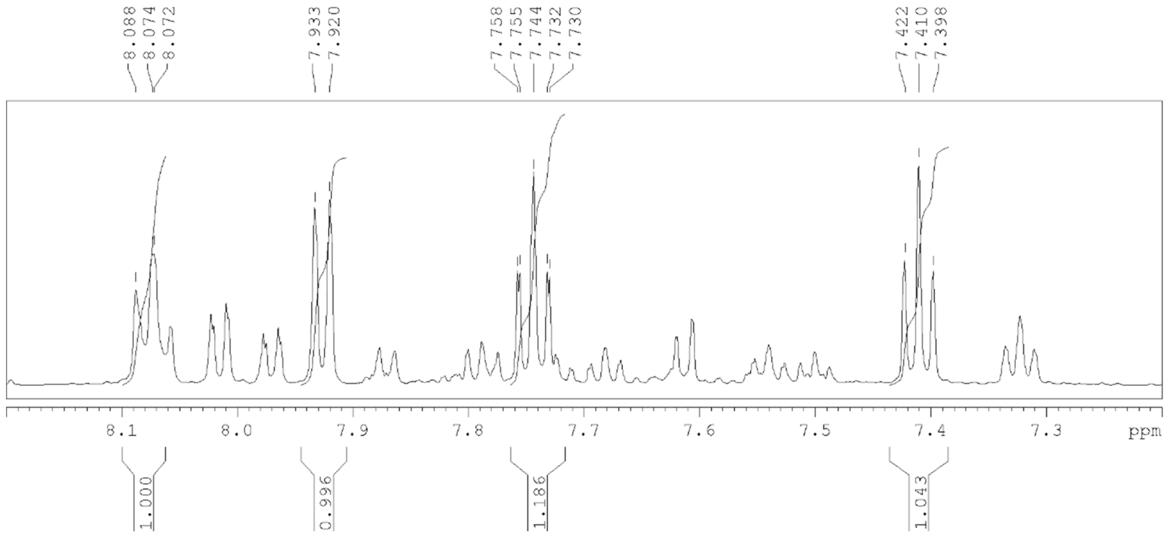

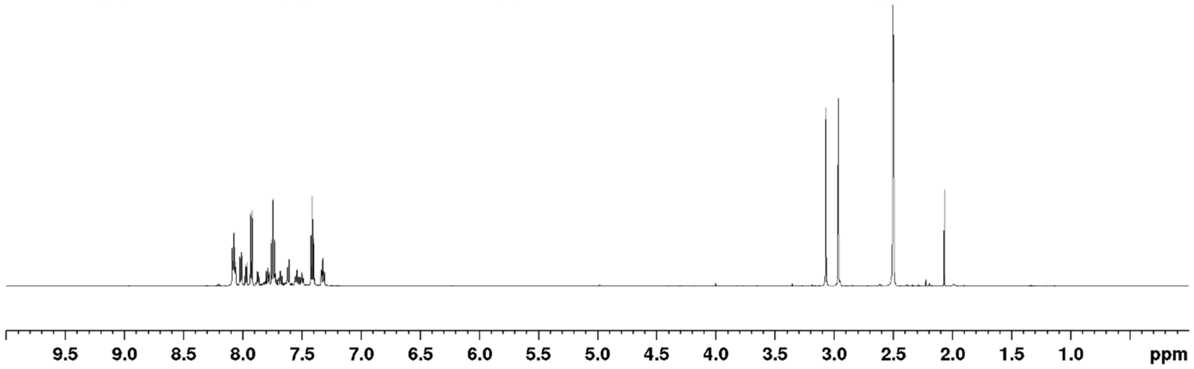

2-(Chlorosulfenyl)benzoyl chloride (4)

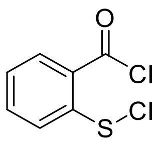

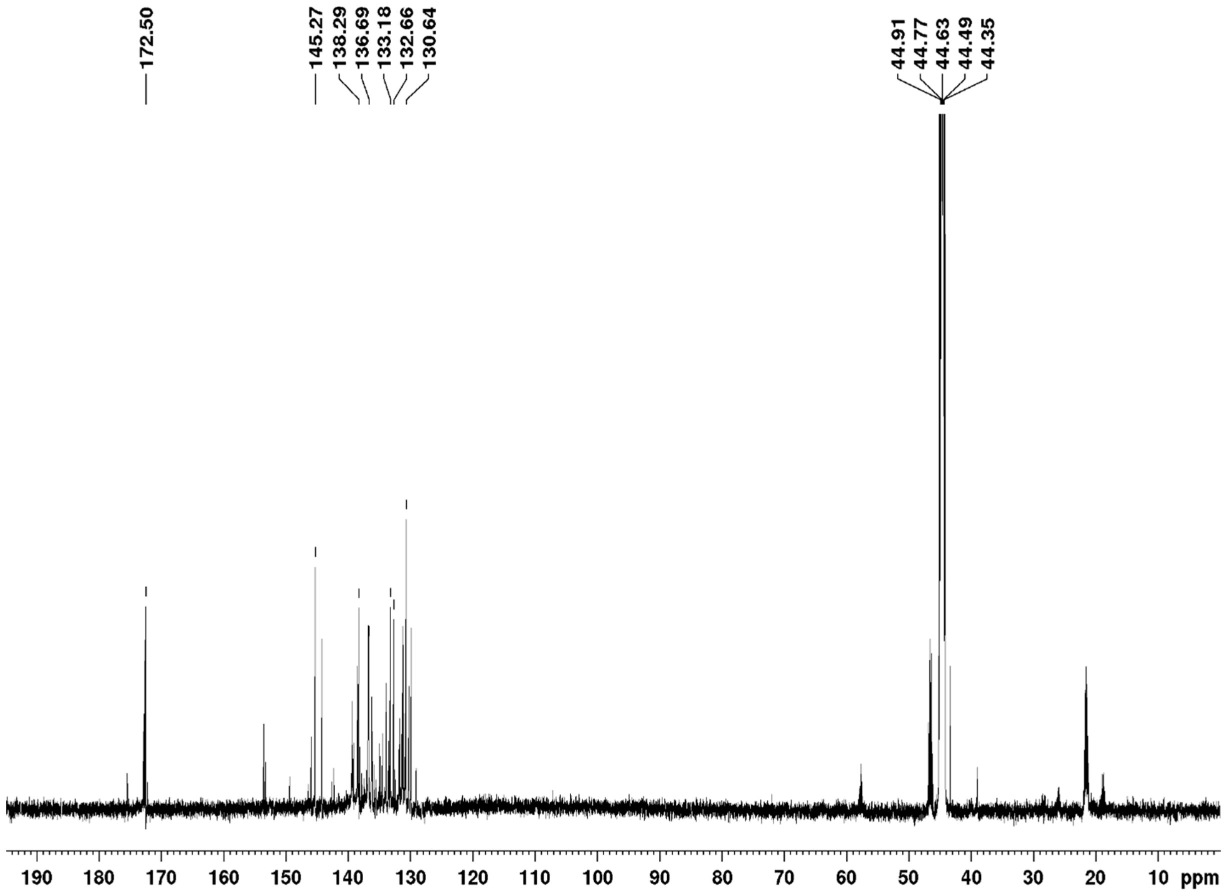

2-Mercaptobenzoyl chloride (6)

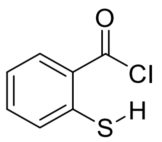

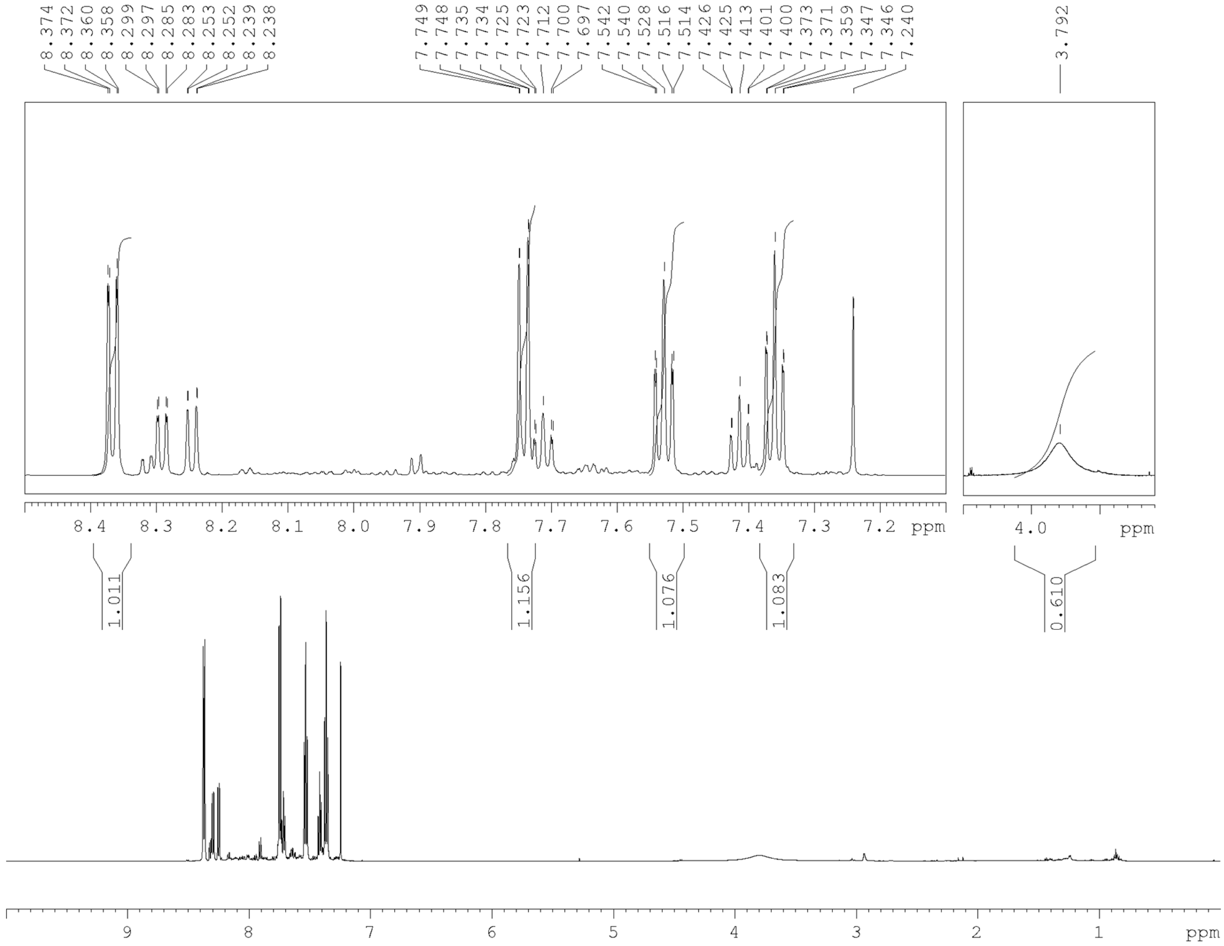

2-Mercaptobenzoyl chloride (6)

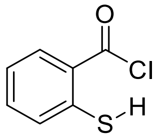

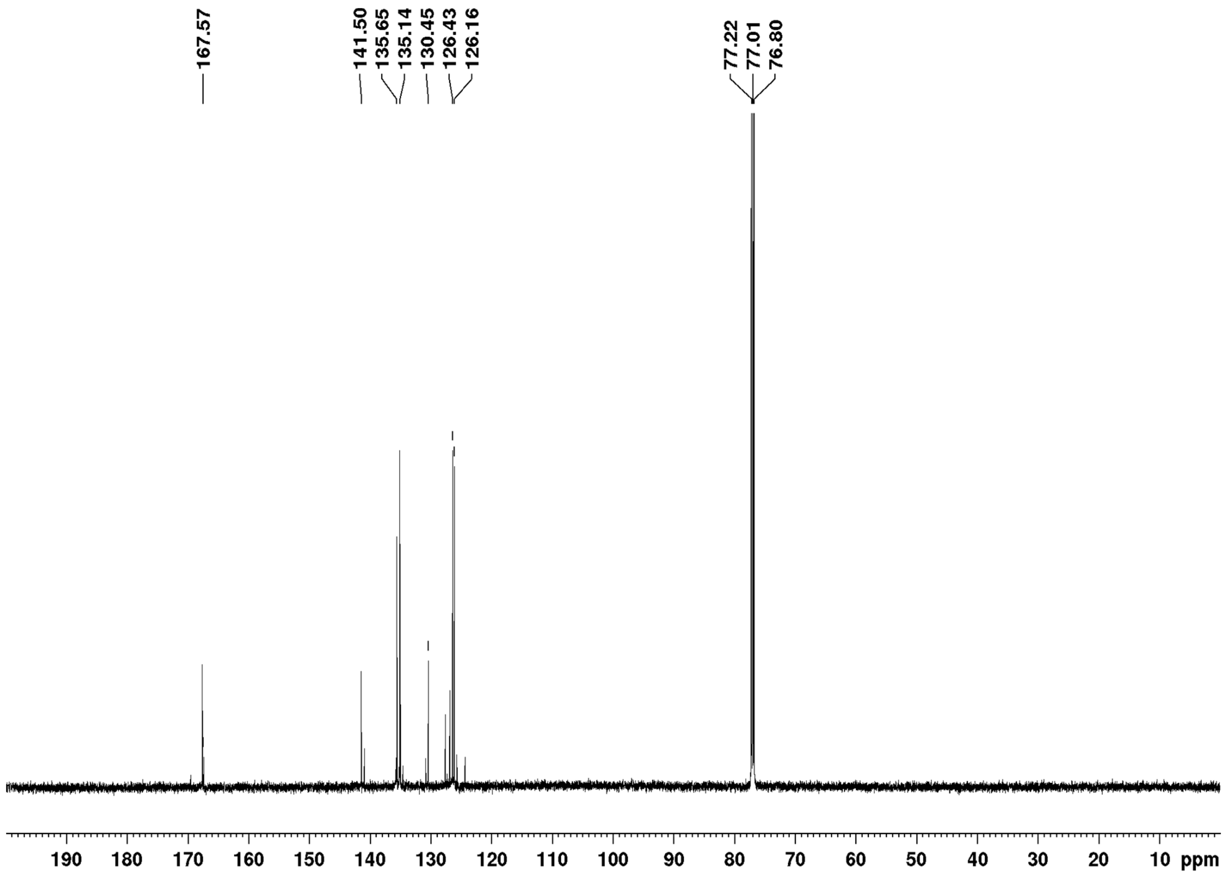

^{1}H & ^{13}C NMR Spectra of 1,2-benzisothiazol-3(2*H*)-ones

(5a)

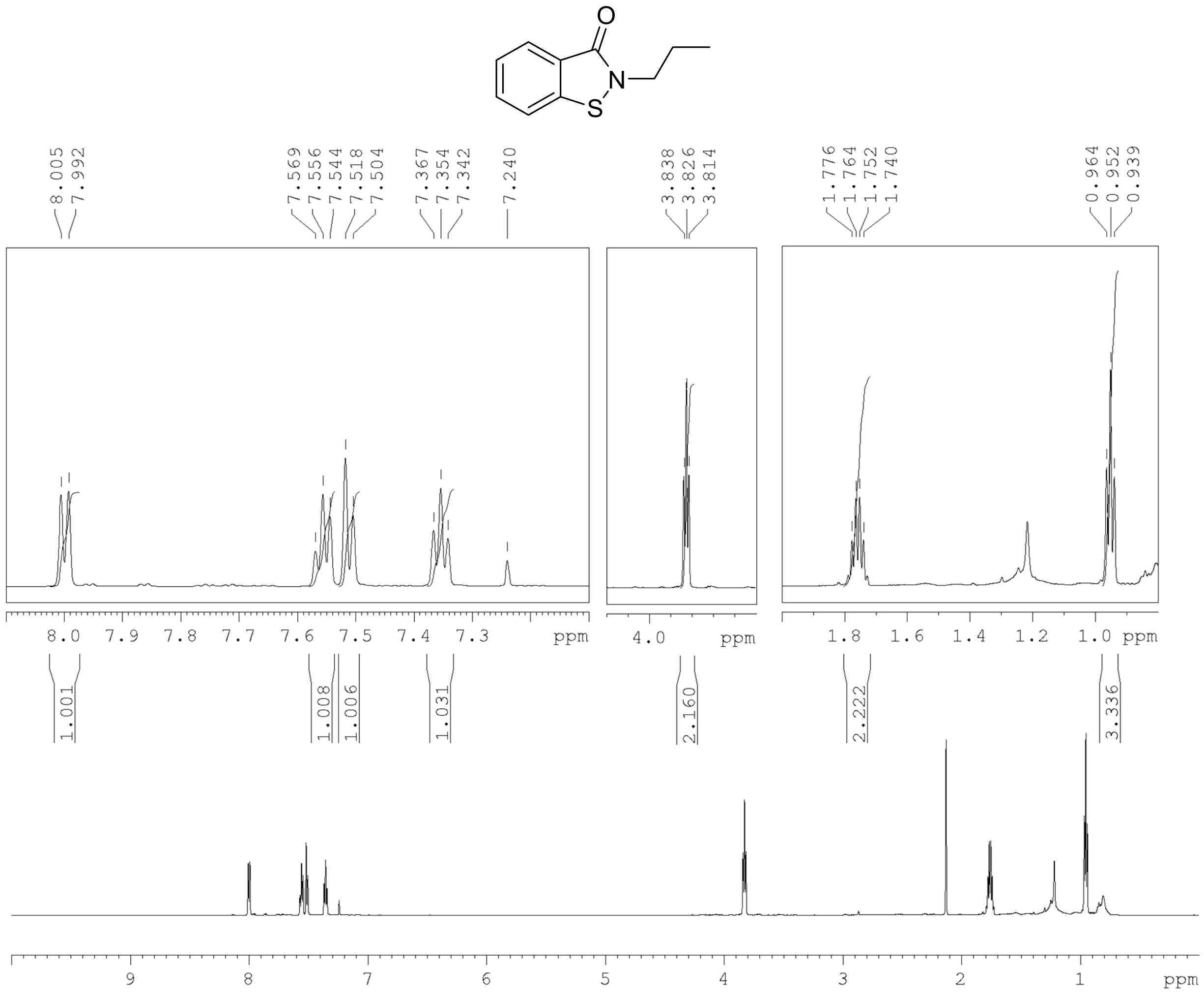

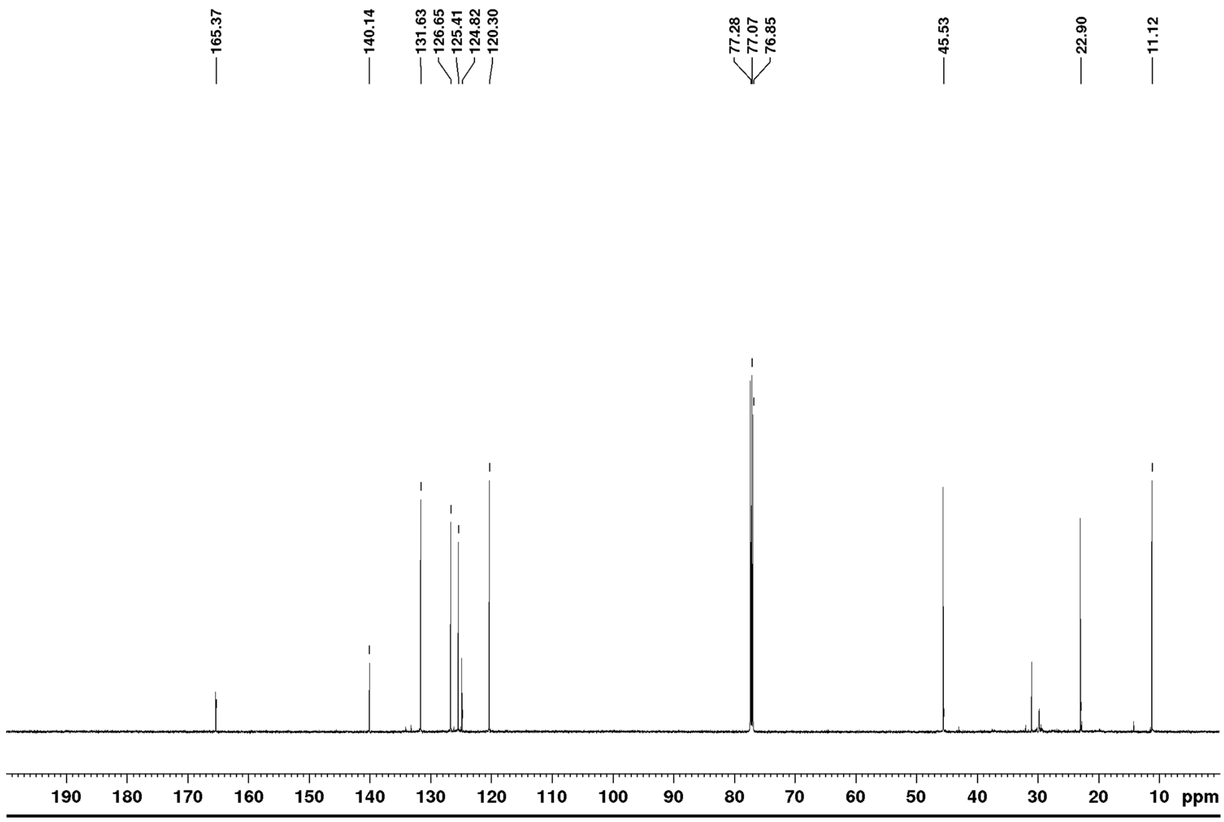
165.37
140.14
131.63
126.65
125.41
124.82
120.30
77.28
77.07
76.85
45.53
22.90
11.12
190 180 170 160 150 140 130 120 110 100 90 80 70 60 50 40 30 20 10 ppm

(5b)

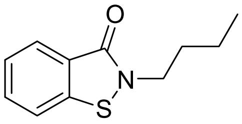
O
N
S

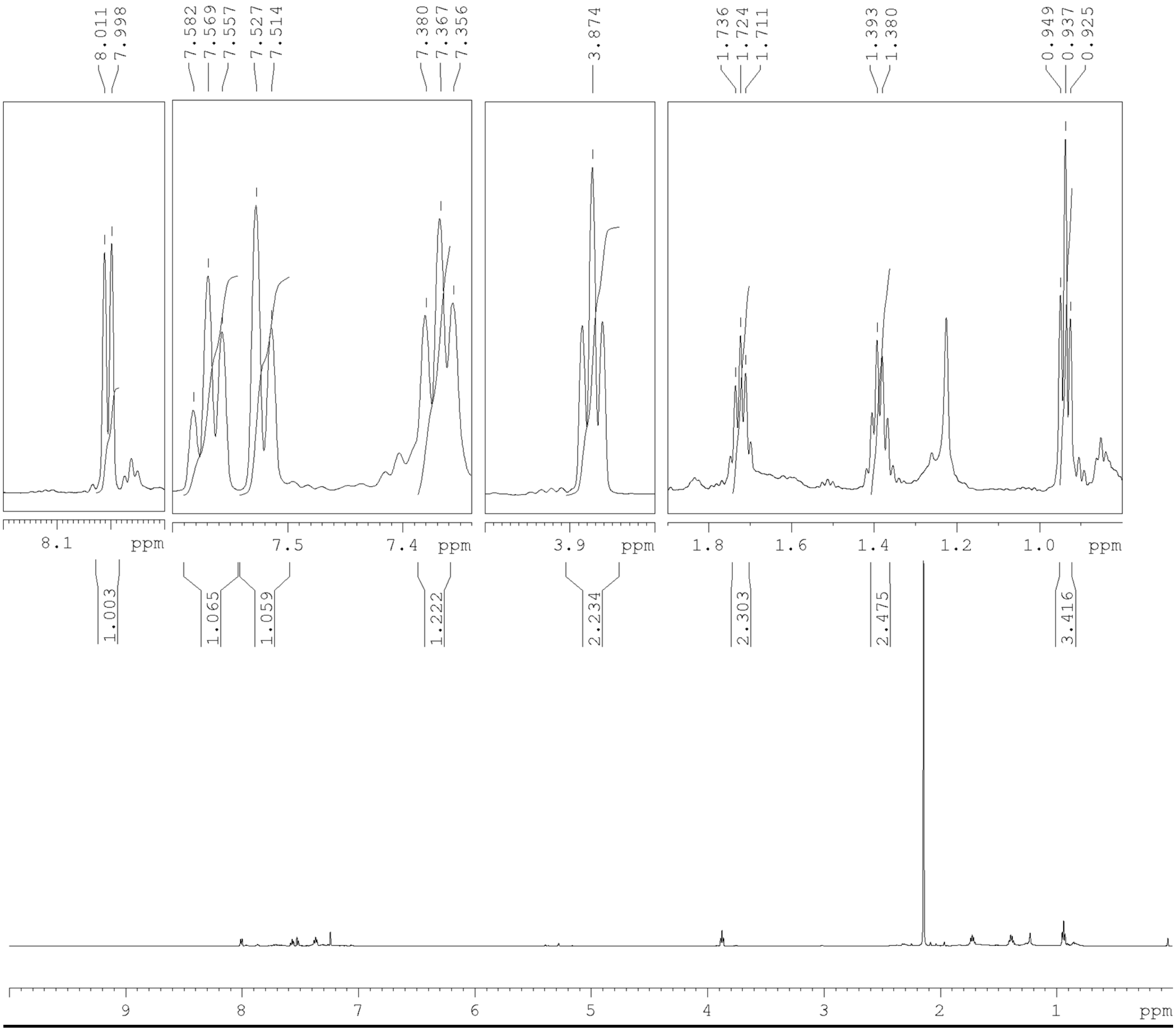
8.011
7.998
7.582
7.569
7.557
7.527
7.514
7.380
7.367
7.356
3.874
1.736
1.724
1.711
1.393
1.380
0.949
0.937
0.925
8.1 ppm
7.5
7.4 ppm
3.9 ppm
1.8 1.6 1.4 1.2 1.0 ppm
1.003
1.065
1.059
1.222
2.234
2.303
2.475
3.416
9 8 7 6 5 4 3 2 1 ppm

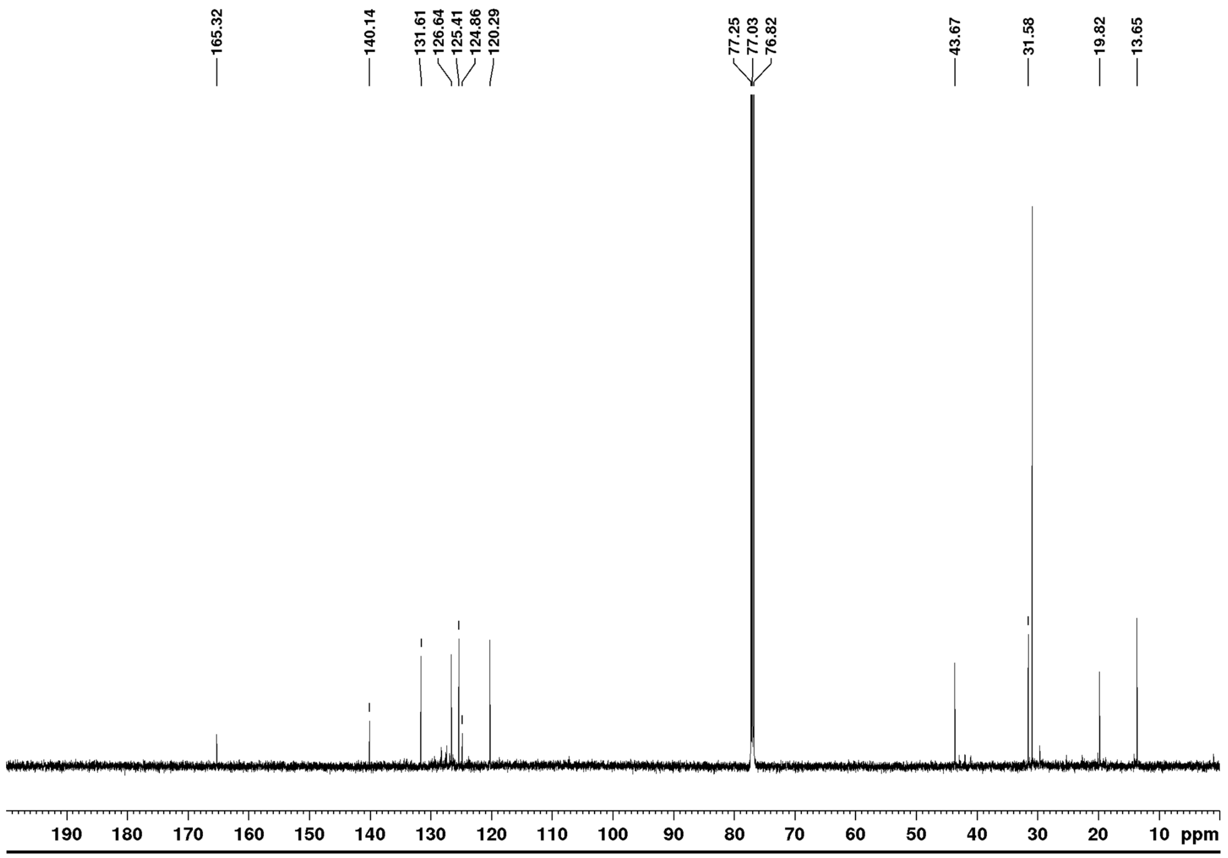
165.32
140.14
131.61
126.64
125.41
124.86
120.29
77.25
77.03
76.82
43.67
31.58
19.82
13.65
190 180 170 160 150 140 130 120 110 100 90 80 70 60 50 40 30 20 10 ppm

(5c)

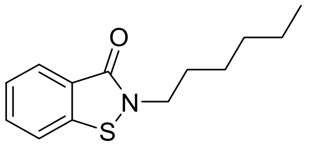
O
N
S

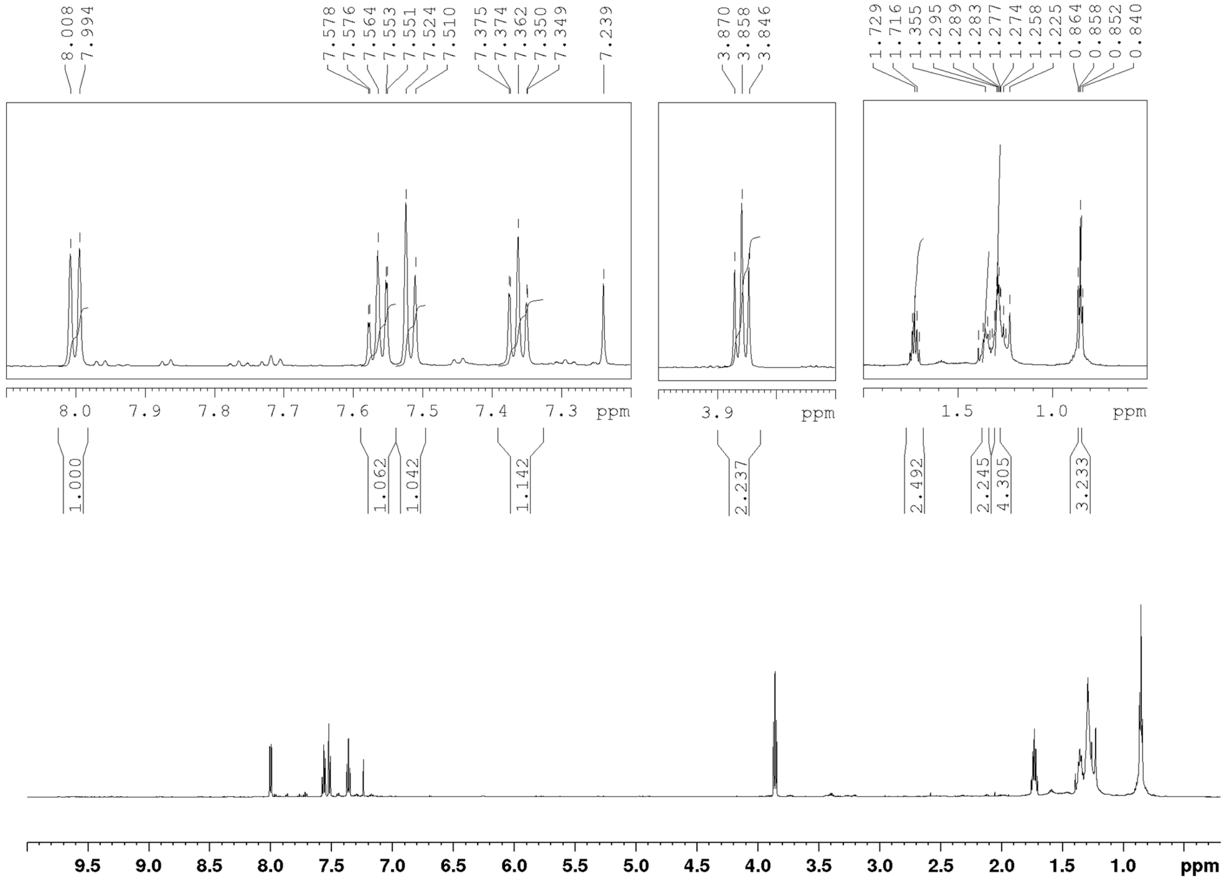
8.008
7.994
7.578
7.576
7.564
7.553
7.551
7.524
7.510
7.375
7.374
7.362
7.350
7.349
7.239
3.870
3.858
3.846
1.729
1.716
1.355
1.295
1.289
1.283
1.277
1.274
1.258
1.225
0.864
0.858
0.852
0.840
8.0 7.9 7.8 7.7 7.6 7.5 7.4 7.3 ppm
3.9 ppm
1.5 1.0 ppm
1.000
1.062
1.042
1.142
2.237
2.492
2.245
4.305
3.233
9.5 9.0 8.5 8.0 7.5 7.0 6.5 6.0 5.5 5.0 4.5 4.0 3.5 3.0 2.5 2.0 1.5 1.0 ppm

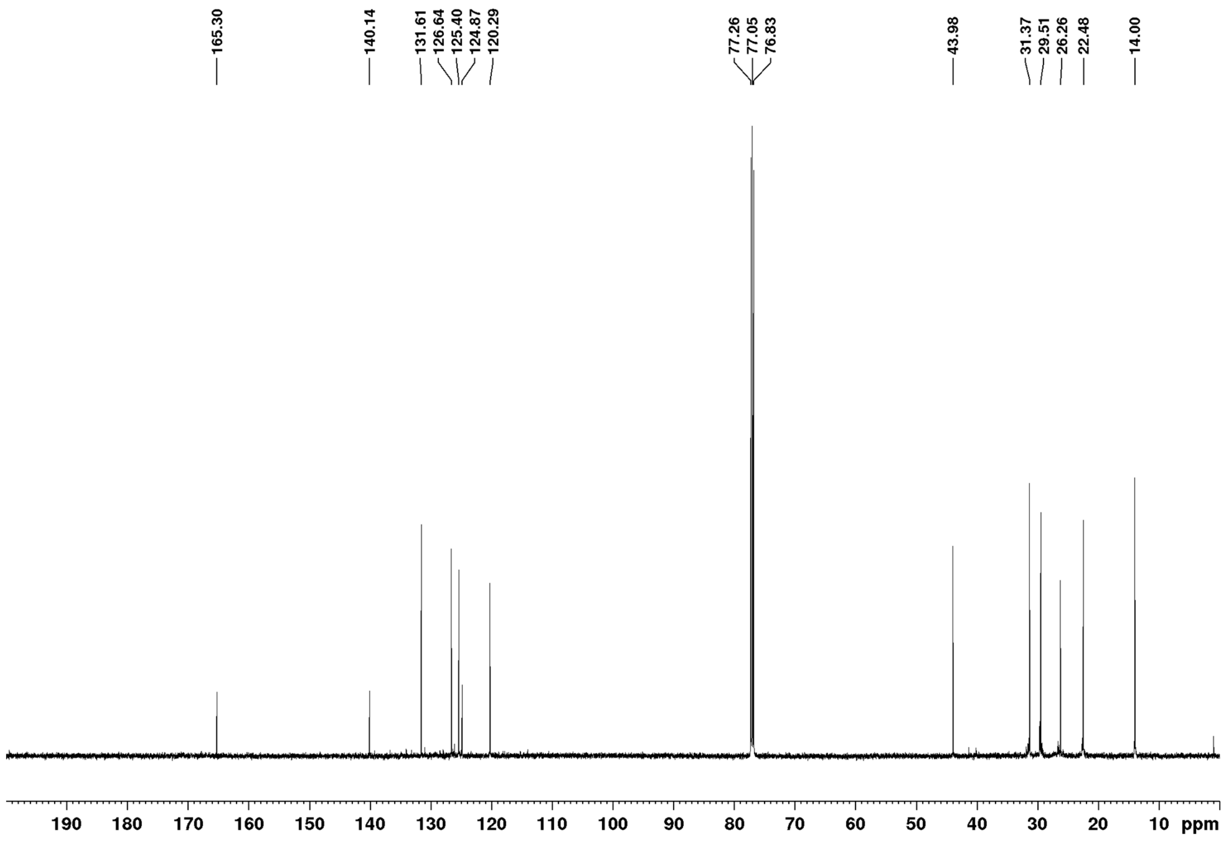
165.30
140.14
131.61
126.64
125.40
124.87
120.29
77.26
77.05
76.83
43.98
31.37
29.51
26.26
22.48
14.00
190 180 170 160 150 140 130 120 110 100 90 80 70 60 50 40 30 20 10 ppm

(5d)

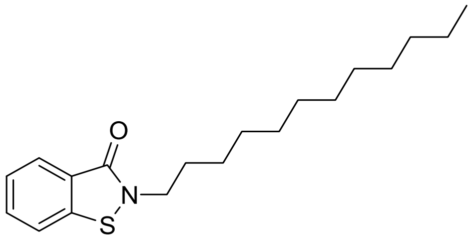

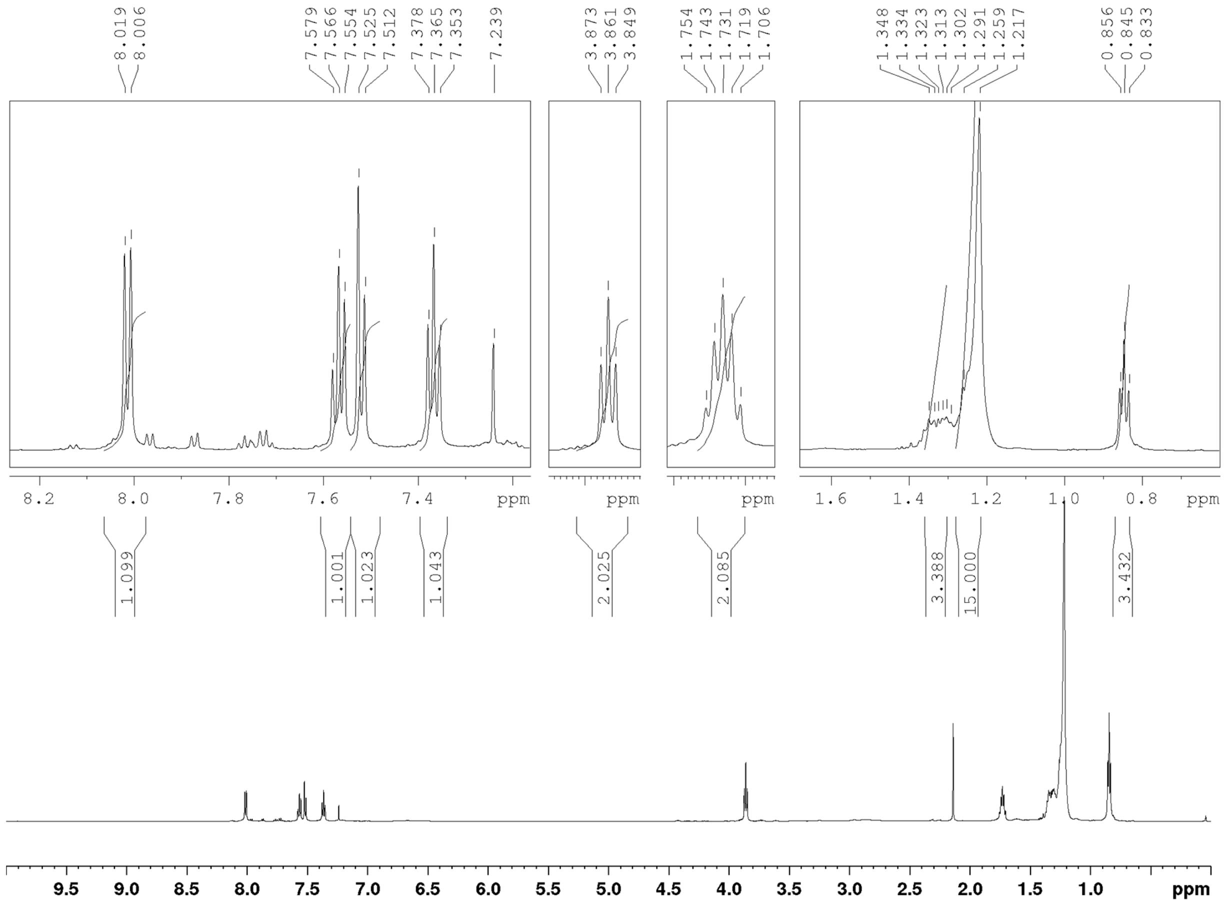
8.019
8.006
7.579
7.566
7.554
7.525
7.512
7.378
7.365
7.353
7.239
3.873
3.861
3.849
1.754
1.743
1.731
1.719
1.706
1.348
1.334
1.323
1.313
1.302
1.291
1.259
1.217
0.856
0.845
0.833
1.099
1.001
1.023
1.043
2.025
2.085
3.388
15.000
3.432
ppm

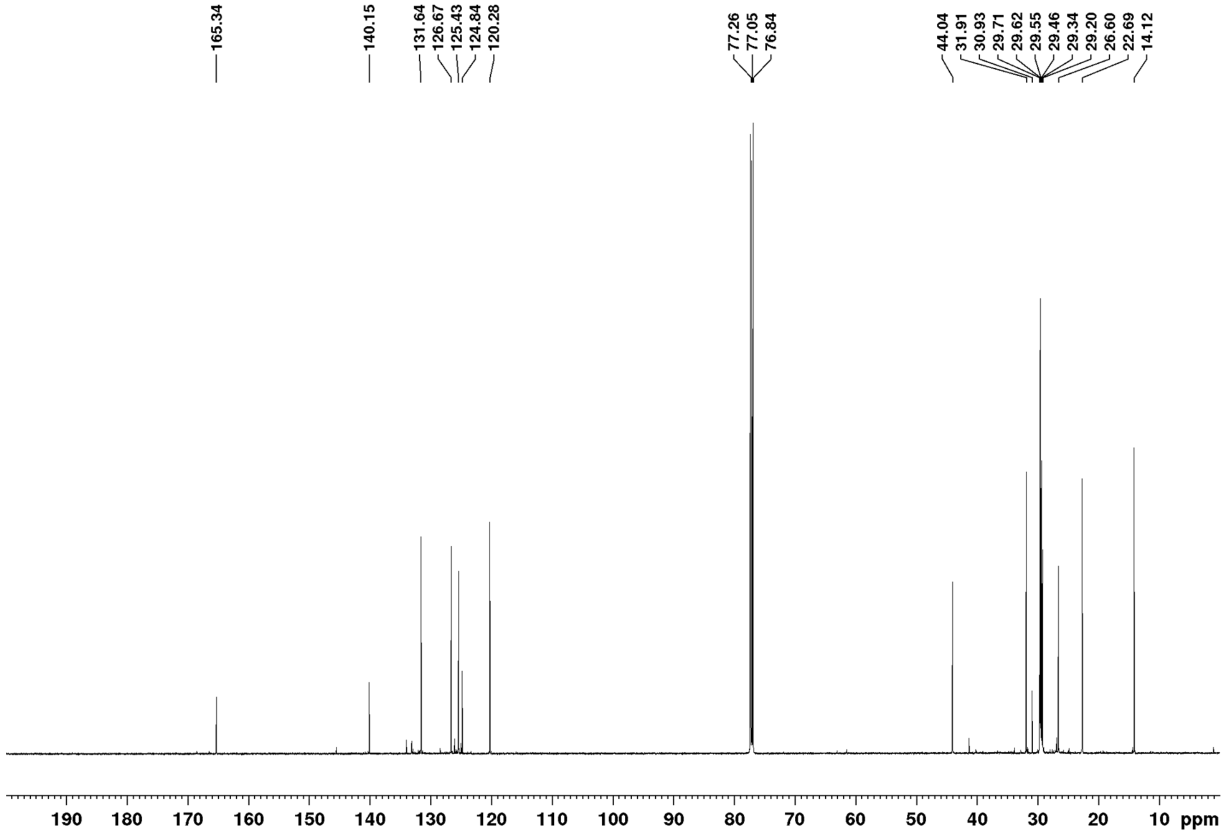

165.34
140.15
131.64
126.67
125.43
124.84
120.28
77.26
77.05
76.84
44.04
31.91
30.93
29.71
29.62
29.55
29.46
29.34
29.20
26.60
22.69
14.12
190 180 170 160 150 140 130 120 110 100 90 80 70 60 50 40 30 20 10 ppm

(5e)

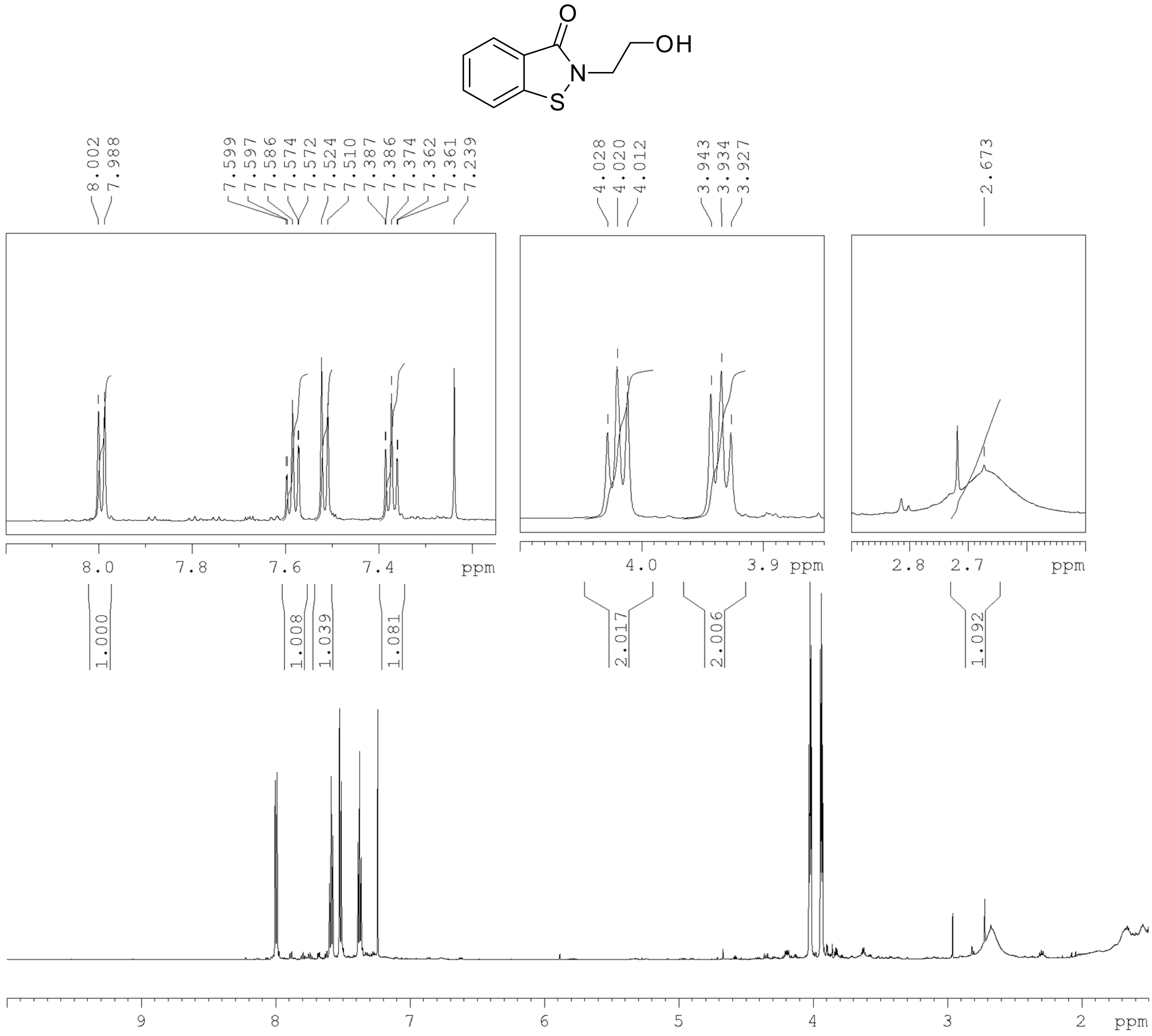
O
N
OH
S
8.002
7.988
7.599
7.597
7.586
7.574
7.572
7.524
7.510
7.387
7.386
7.374
7.362
7.361
7.239
4.028
4.020
4.012
3.943
3.934
3.927
2.673
8.0
7.8
7.6
7.4
ppm
4.0
3.9 ppm
2.8
2.7
ppm
1.000
1.008
1.039
1.081
2.017
2.006
1.092
9
8
7
6
5
4
3
2
ppm

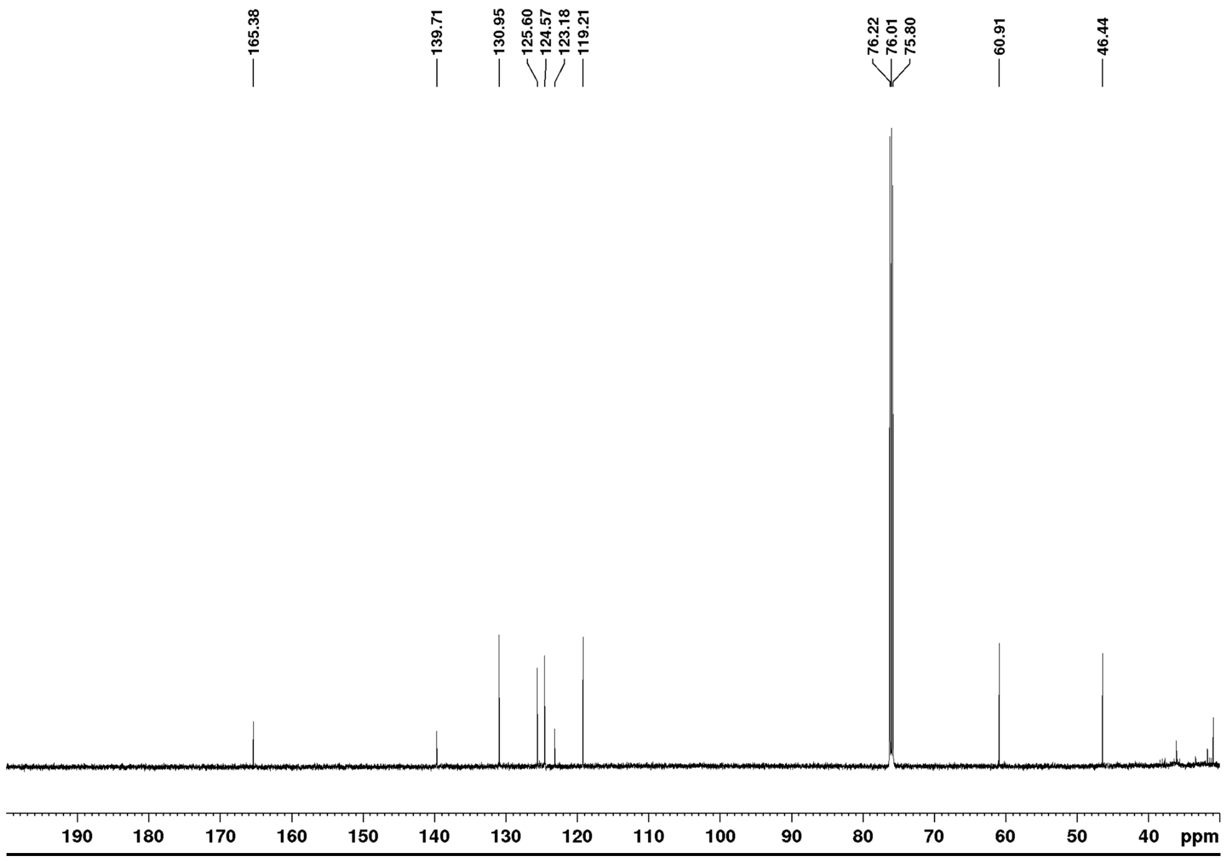
165.38
139.71
130.95
125.60
124.57
123.18
119.21
76.22
76.01
75.80
60.91
46.44
190 180 170 160 150 140 130 120 110 100 90 80 70 60 50 40 ppm

(5f)

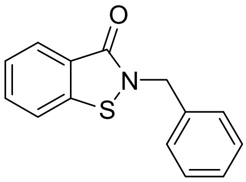
O
N
S

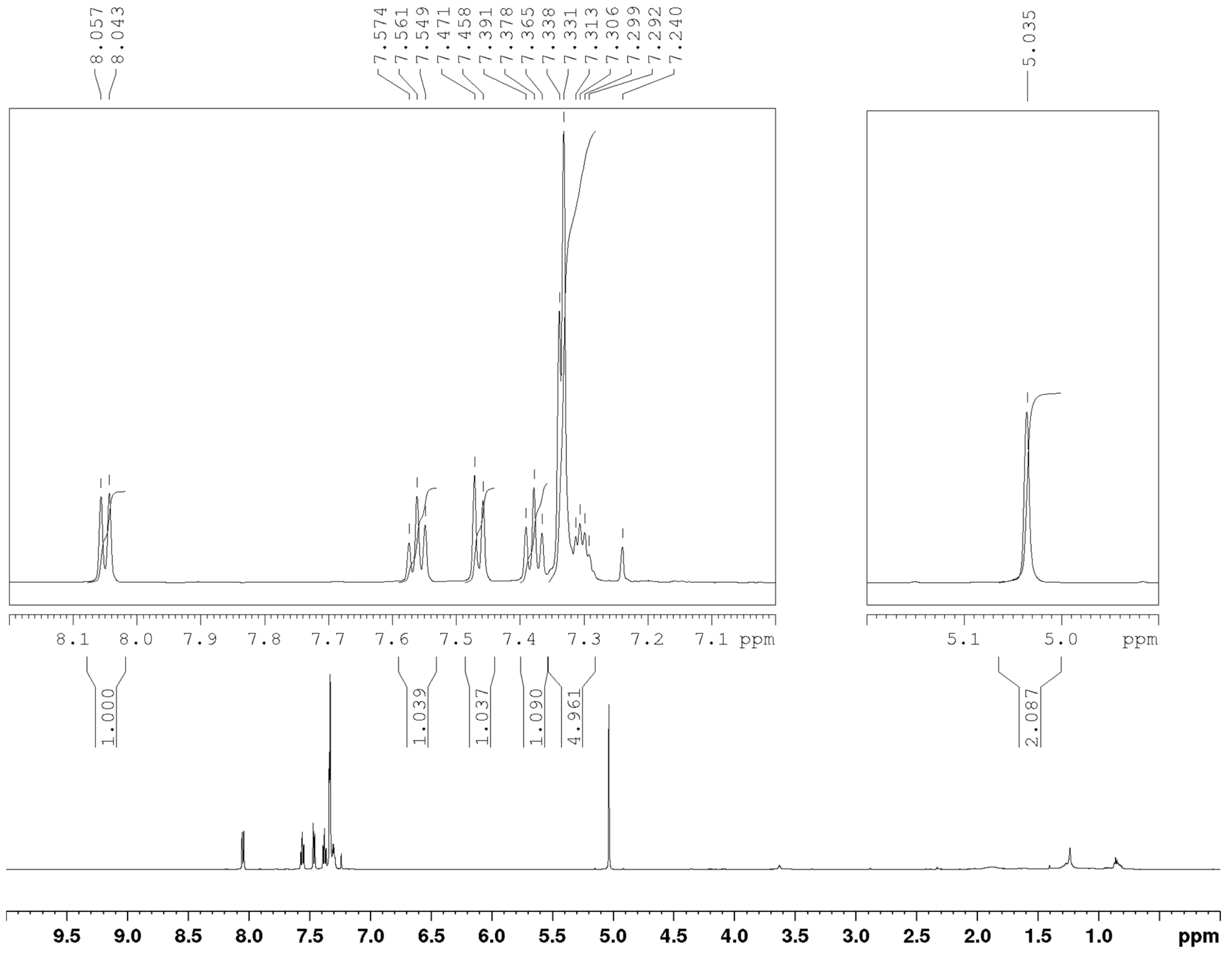
8.057
8.043
7.574
7.561
7.549
7.471
7.458
7.391
7.378
7.365
7.338
7.331
7.313
7.306
7.299
7.292
7.240
5.035
8.1 8.0 7.9 7.8 7.7 7.6 7.5 7.4 7.3 7.2 7.1 ppm
5.1 5.0 ppm
1.000
1.039
1.037
1.090
4.961
2.087
9.5 9.0 8.5 8.0 7.5 7.0 6.5 6.0 5.5 5.0 4.5 4.0 3.5 3.0 2.5 2.0 1.5 1.0 ppm

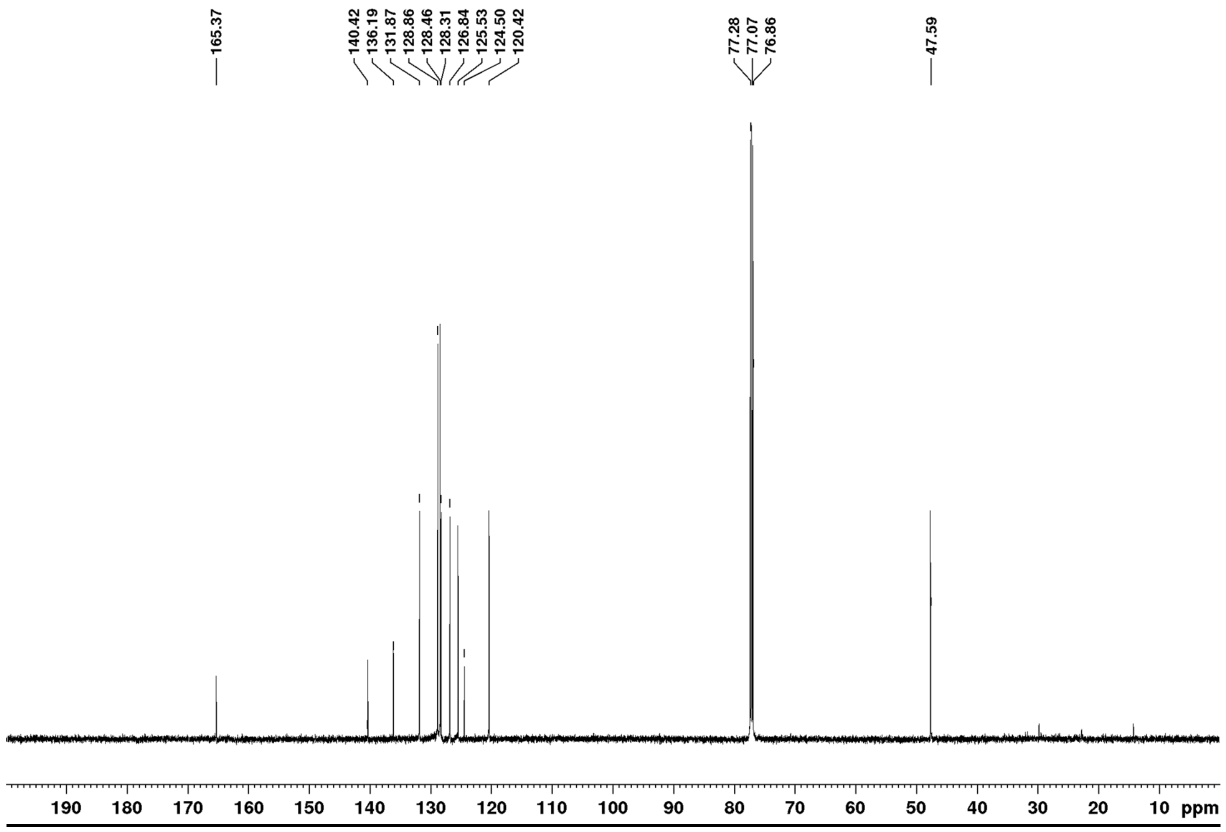
165.37
140.42
136.19
131.87
128.86
128.46
128.31
126.84
125.53
124.50
120.42
77.28
77.07
76.86
47.59
190 180 170 160 150 140 130 120 110 100 90 80 70 60 50 40 30 20 10 ppm

(5g)

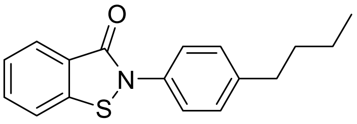
O
N
S

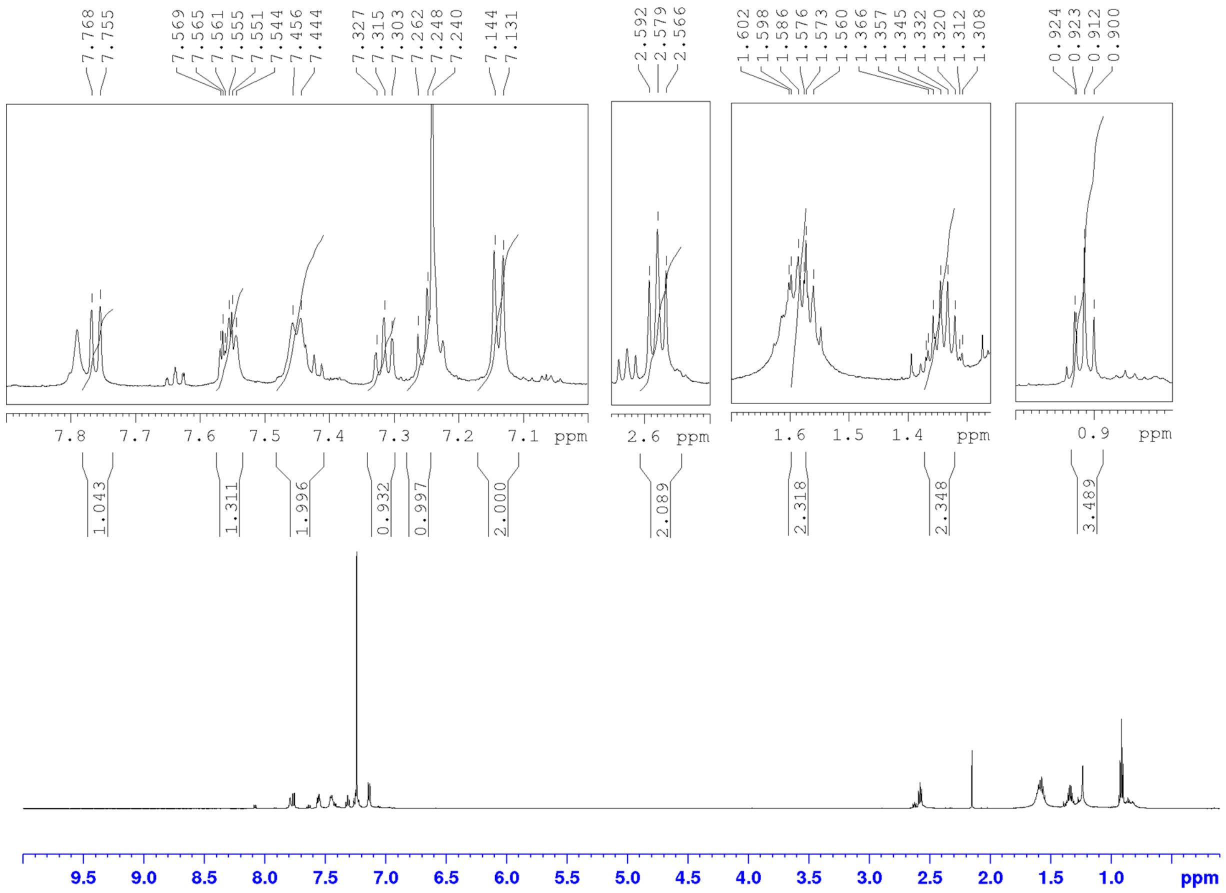
7.768
7.755
7.569
7.565
7.561
7.555
7.551
7.544
7.456
7.444
7.327
7.315
7.303
7.262
7.248
7.240
7.144
7.131
2.592
2.579
2.566
1.602
1.598
1.586
1.576
1.573
1.560
1.366
1.357
1.345
1.332
1.320
1.312
1.308
0.924
0.923
0.912
0.900
1.043
1.311
1.996
0.932
0.997
2.000
2.089
2.318
2.348
3.489
ppm

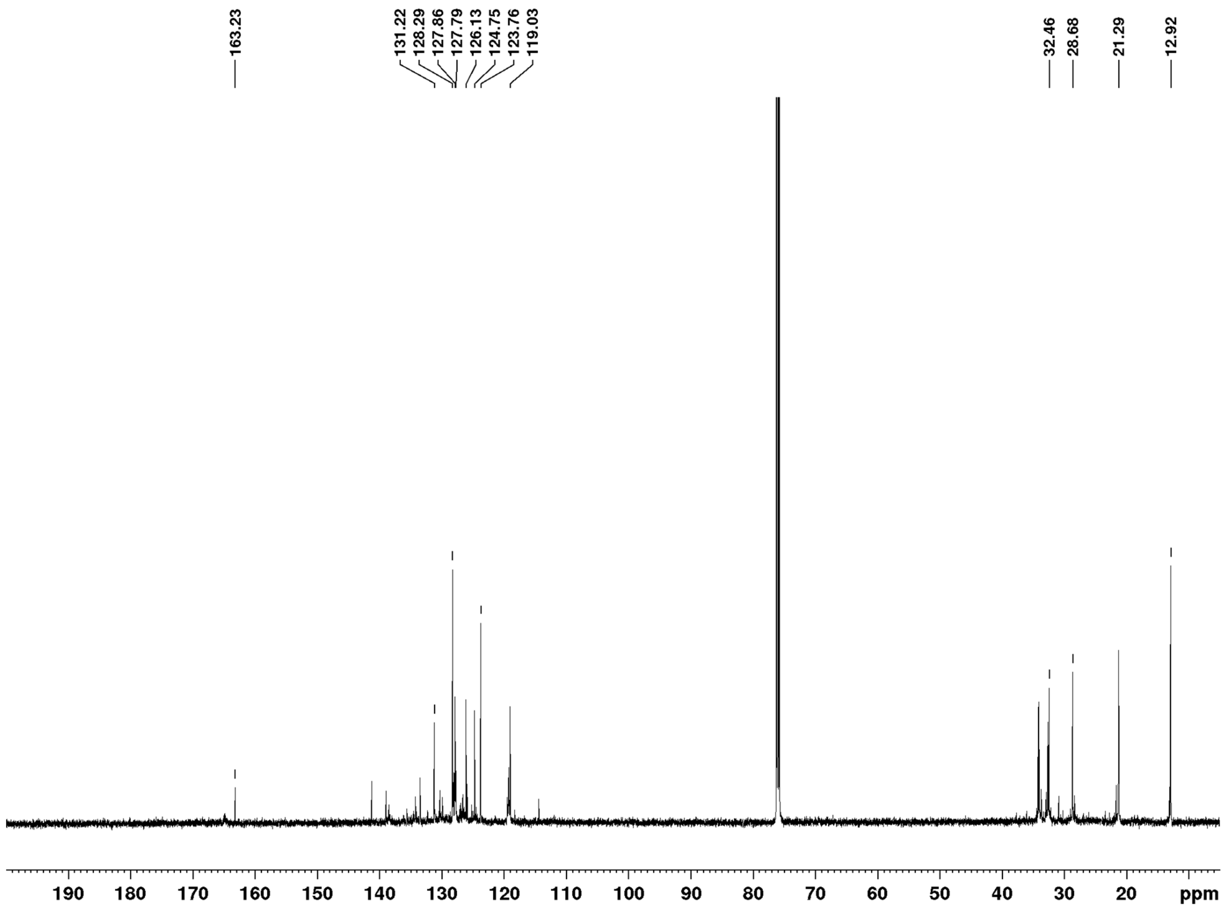
163.23
131.22
128.29
127.86
127.79
126.13
124.75
123.76
119.03
32.46
28.68
21.29
12.92
190
180
170
160
150
140
130
120
110
100
90
80
70
60
50
40
30
20
ppm

(5h)

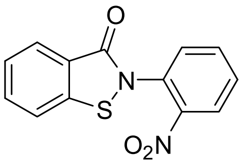
O
N
S
O2N

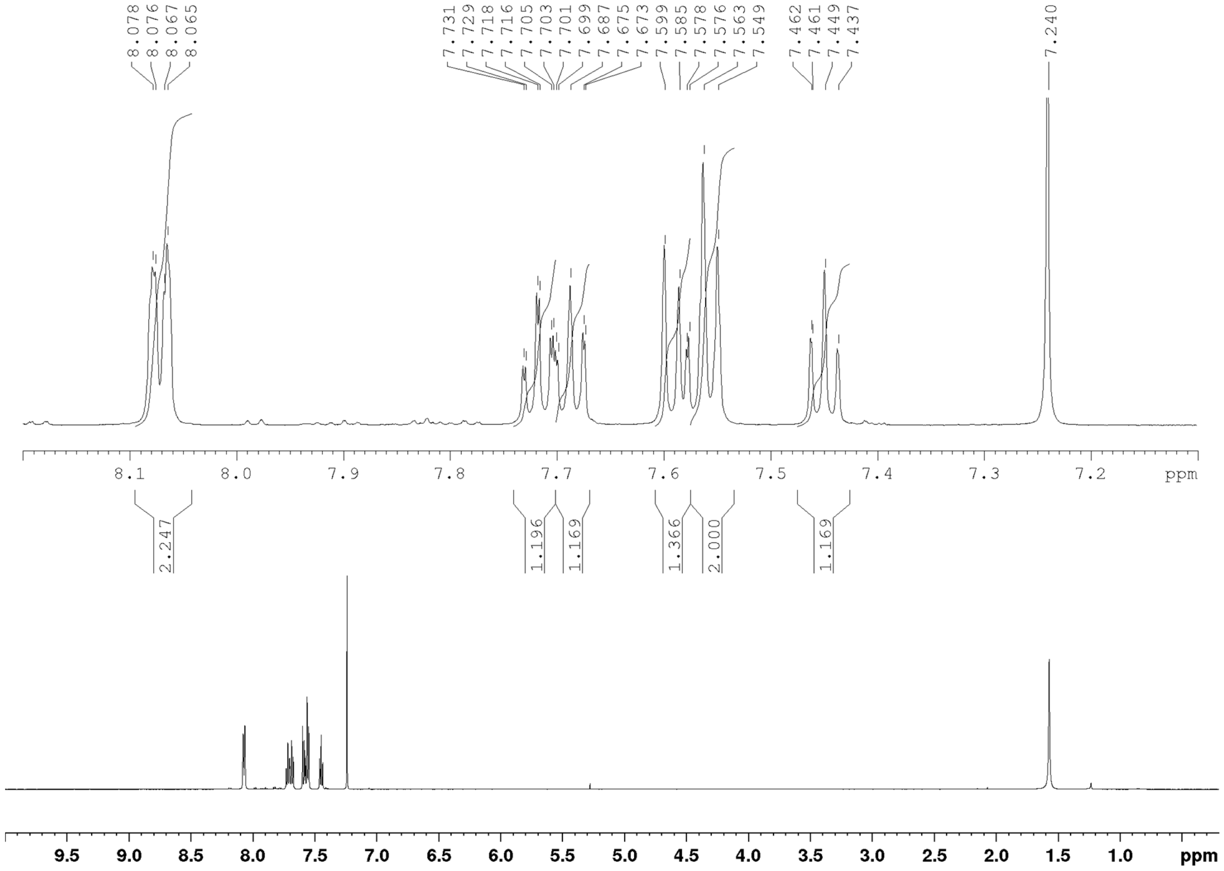
8.078
8.076
8.067
8.065
7.731
7.729
7.718
7.716
7.705
7.703
7.701
7.699
7.687
7.675
7.673
7.599
7.585
7.578
7.576
7.563
7.549
7.462
7.461
7.449
7.437
7.240
8.1
8.0
7.9
7.8
7.7
7.6
7.5
7.4
7.3
7.2
ppm
2.247
1.196
1.169
1.366
2.000
1.169
9.5
9.0
8.5
8.0
7.5
7.0
6.5
6.0
5.5
5.0
4.5
4.0
3.5
3.0
2.5
2.0
1.5
1.0
ppm

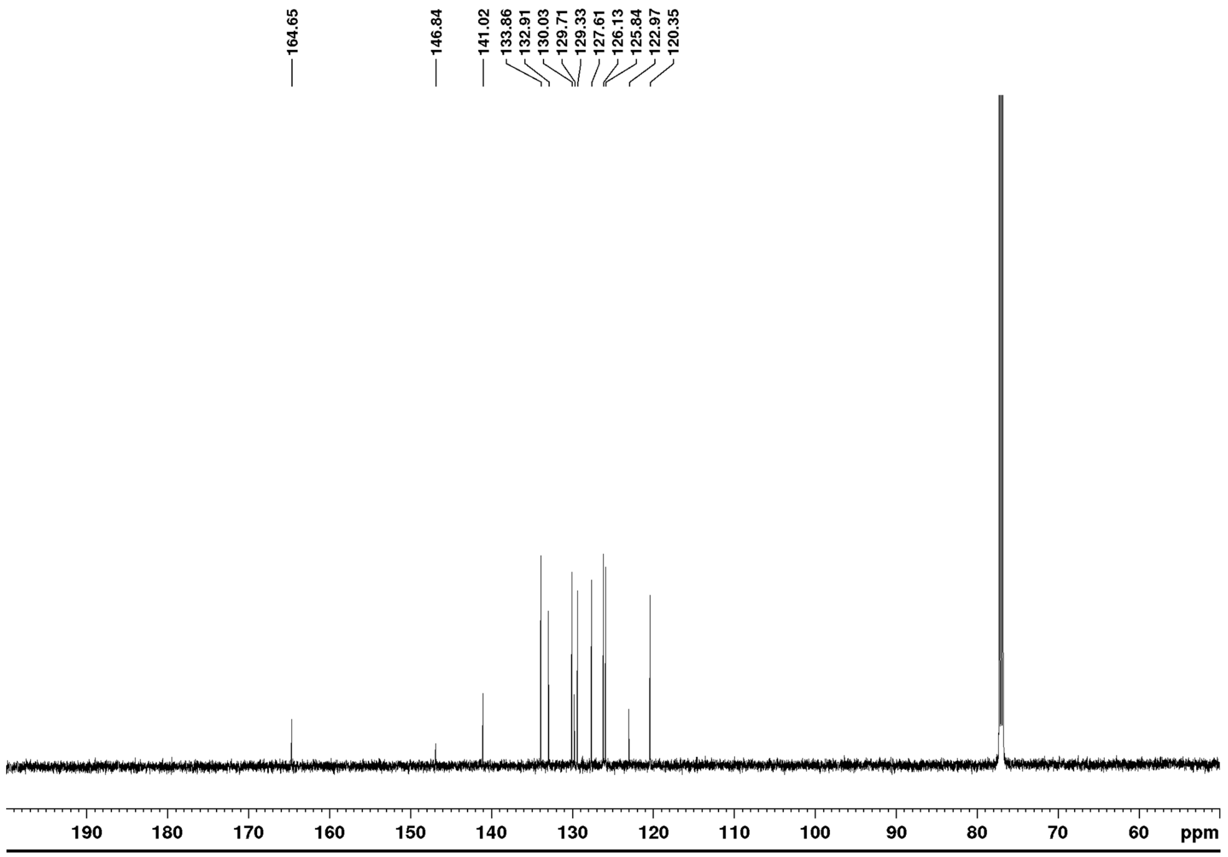
164.65
146.84
141.02
133.86
132.91
130.03
129.71
129.33
127.61
126.13
125.84
122.97
120.35
190 180 170 160 150 140 130 120 110 100 90 80 70 60 ppm

(5i)

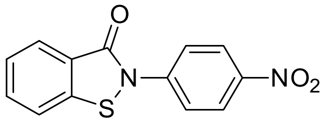

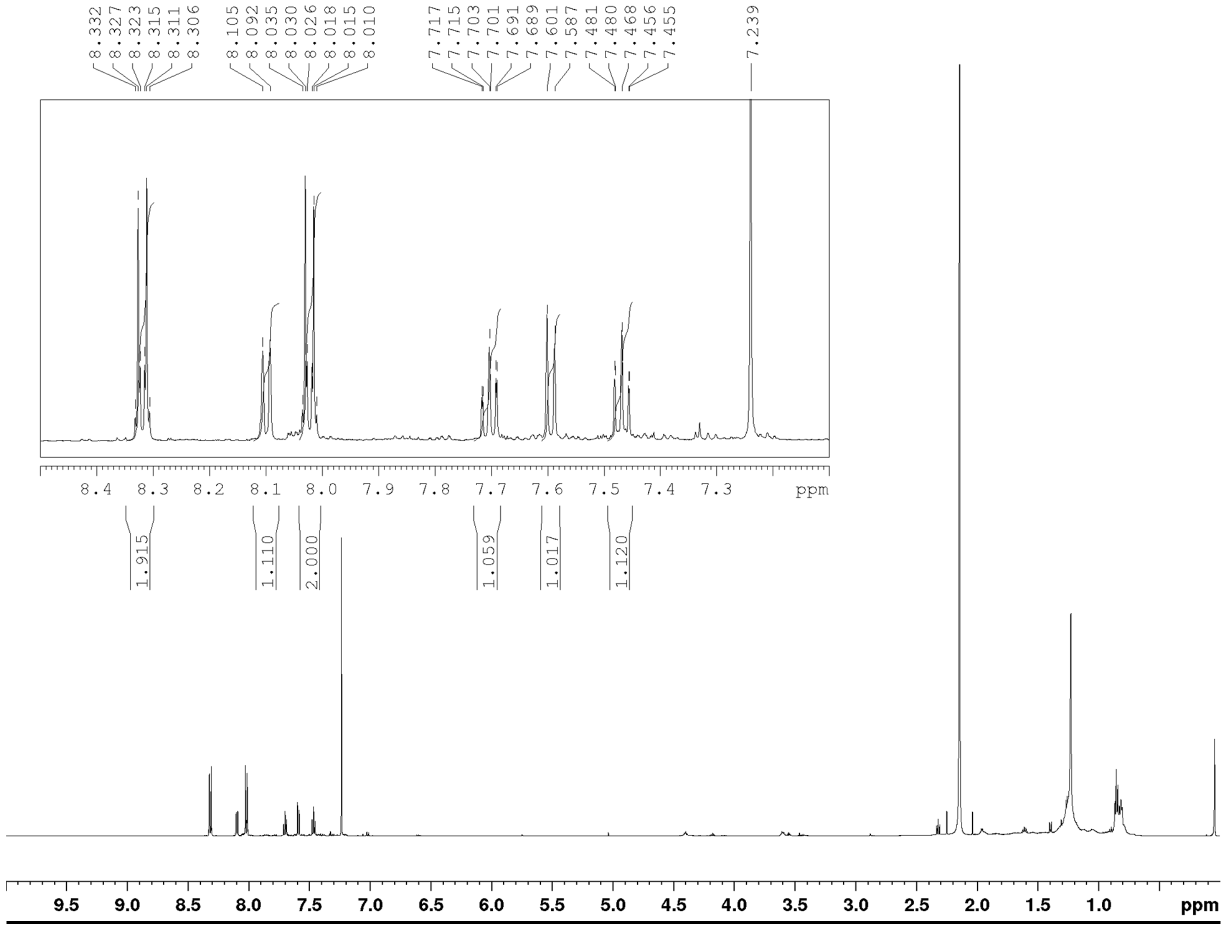

8.332
8.327
8.323
8.315
8.311
8.306
8.105
8.092
8.035
8.030
8.026
8.018
8.015
8.010
7.717
7.715
7.703
7.701
7.691
7.689
7.601
7.587
7.481
7.480
7.468
7.456
7.455
7.239
8.4 8.3 8.2 8.1 8.0 7.9 7.8 7.7 7.6 7.5 7.4 7.3 ppm
1.915
1.110
2.000
1.059
1.017
1.120
9.5 9.0 8.5 8.0 7.5 7.0 6.5 6.0 5.5 5.0 4.5 4.0 3.5 3.0 2.5 2.0 1.5 1.0 ppm

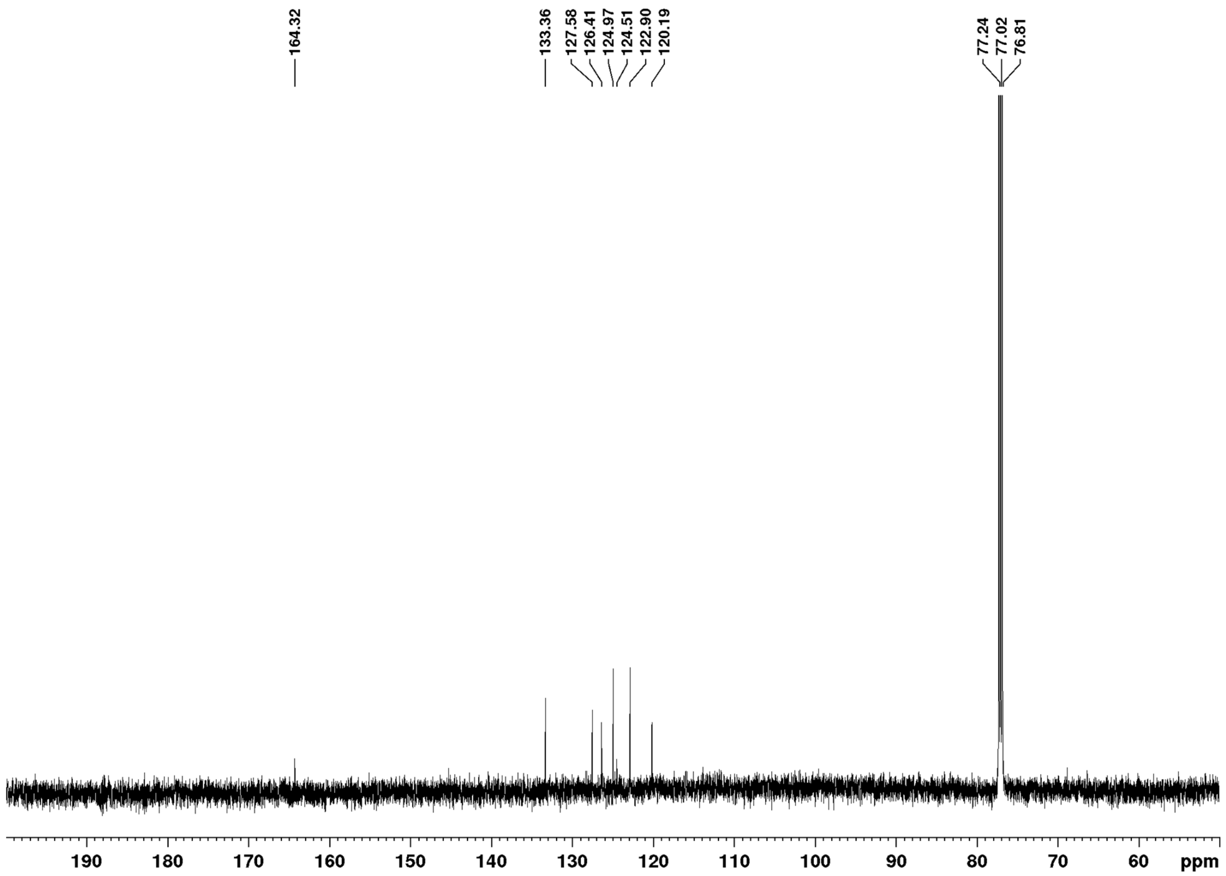
164.32
133.36
127.58
126.41
124.97
124.51
122.90
120.19
77.24
77.02
76.81
190 180 170 160 150 140 130 120 110 100 90 80 70 60 ppm

(5j)

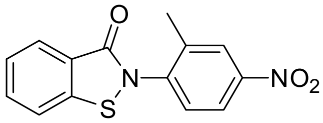

O
N
S
NO2

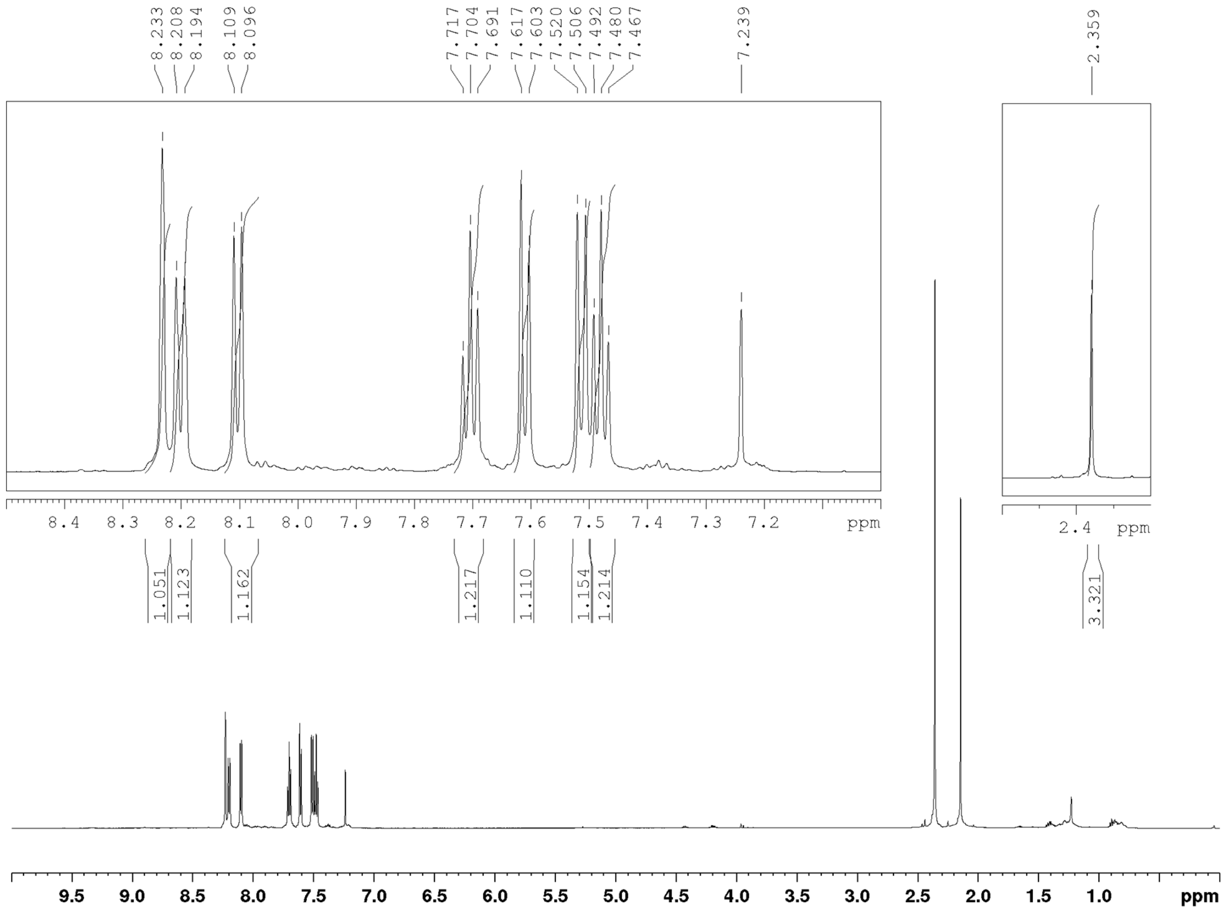

8.233
8.208
8.194
8.109
8.096
7.717
7.704
7.691
7.617
7.603
7.520
7.506
7.492
7.480
7.467
7.239
2.359
8.4 8.3 8.2 8.1 8.0 7.9 7.8 7.7 7.6 7.5 7.4 7.3 7.2 ppm
2.4 ppm
1.051
1.123
1.162
1.217
1.110
1.154
1.214
3.321
9.5 9.0 8.5 8.0 7.5 7.0 6.5 6.0 5.5 5.0 4.5 4.0 3.5 3.0 2.5 2.0 1.5 1.0 ppm

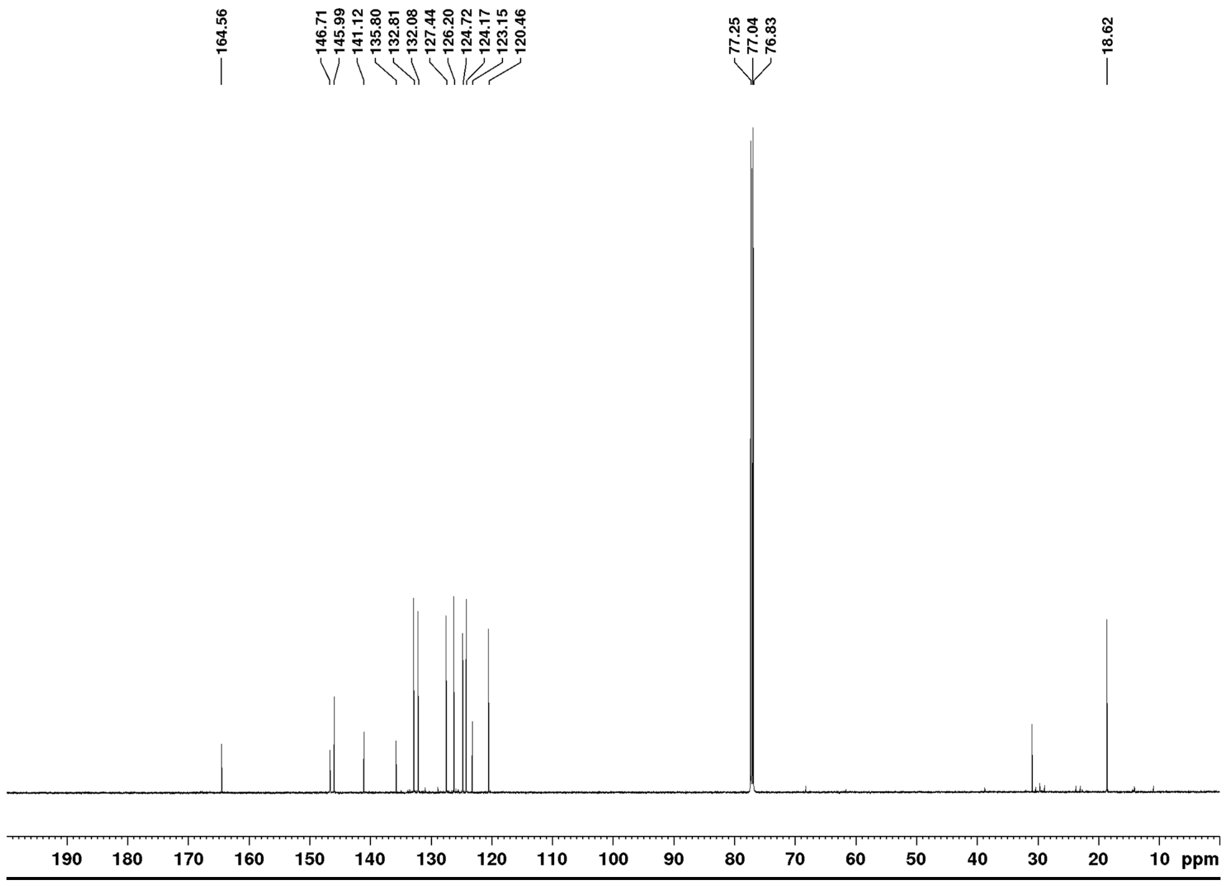
164.56
146.71
145.99
141.12
135.80
132.81
132.08
127.44
126.20
124.72
124.17
123.15
120.46
77.25
77.04
76.83
18.62
190 180 170 160 150 140 130 120 110 100 90 80 70 60 50 40 30 20 10 ppm

(5k)

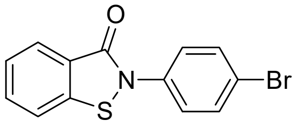
O
N
S
Br

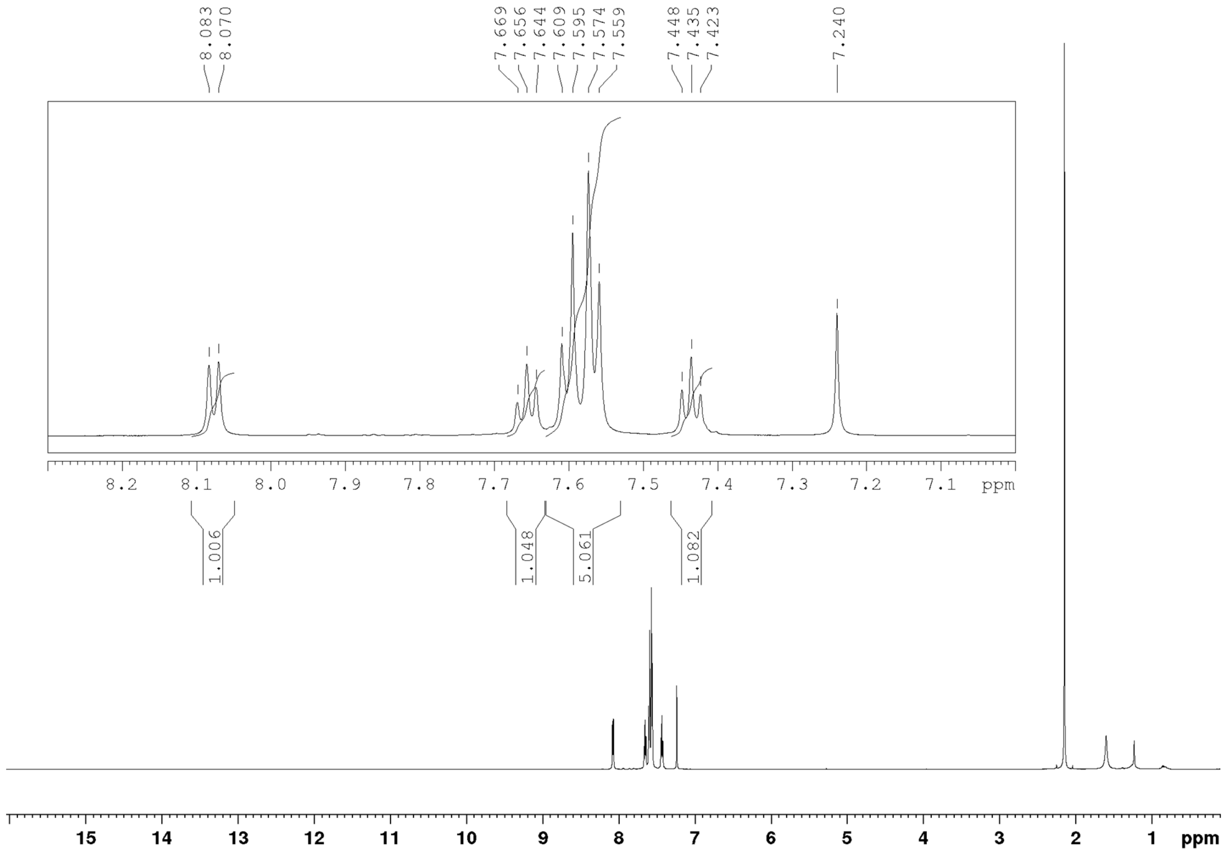
8.083
8.070
7.669
7.656
7.644
7.609
7.595
7.574
7.559
7.448
7.435
7.423
7.240
8.2 8.1 8.0 7.9 7.8 7.7 7.6 7.5 7.4 7.3 7.2 7.1 ppm
1.006
1.048
5.061
1.082
15 14 13 12 11 10 9 8 7 6 5 4 3 2 1 ppm

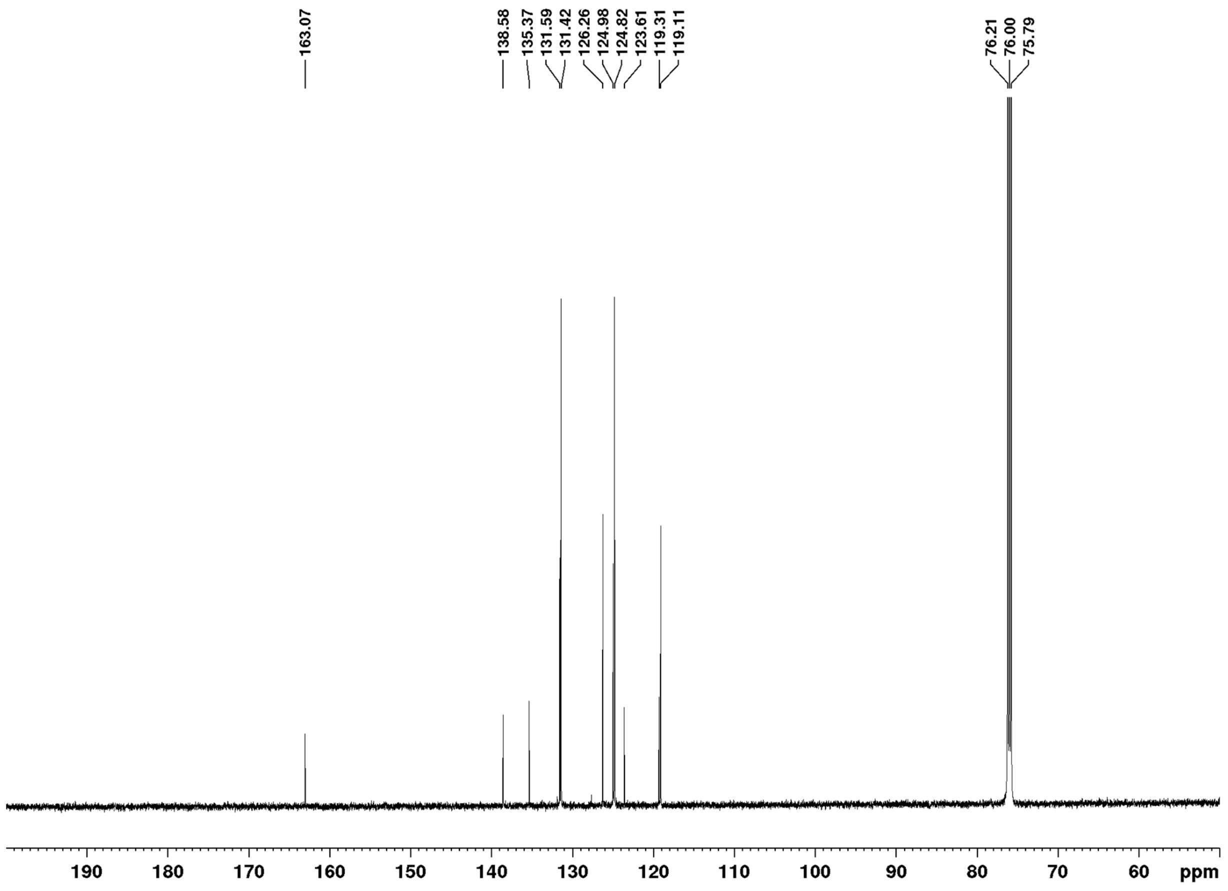
163.07
138.58
135.37
131.59
131.42
126.26
124.98
124.82
123.61
119.31
119.11
76.21
76.00
75.79
190
180
170
160
150
140
130
120
110
100
90
80
70
60
ppm

(5l)

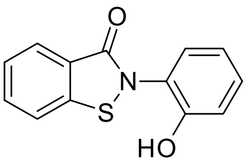
O
N
S
HO

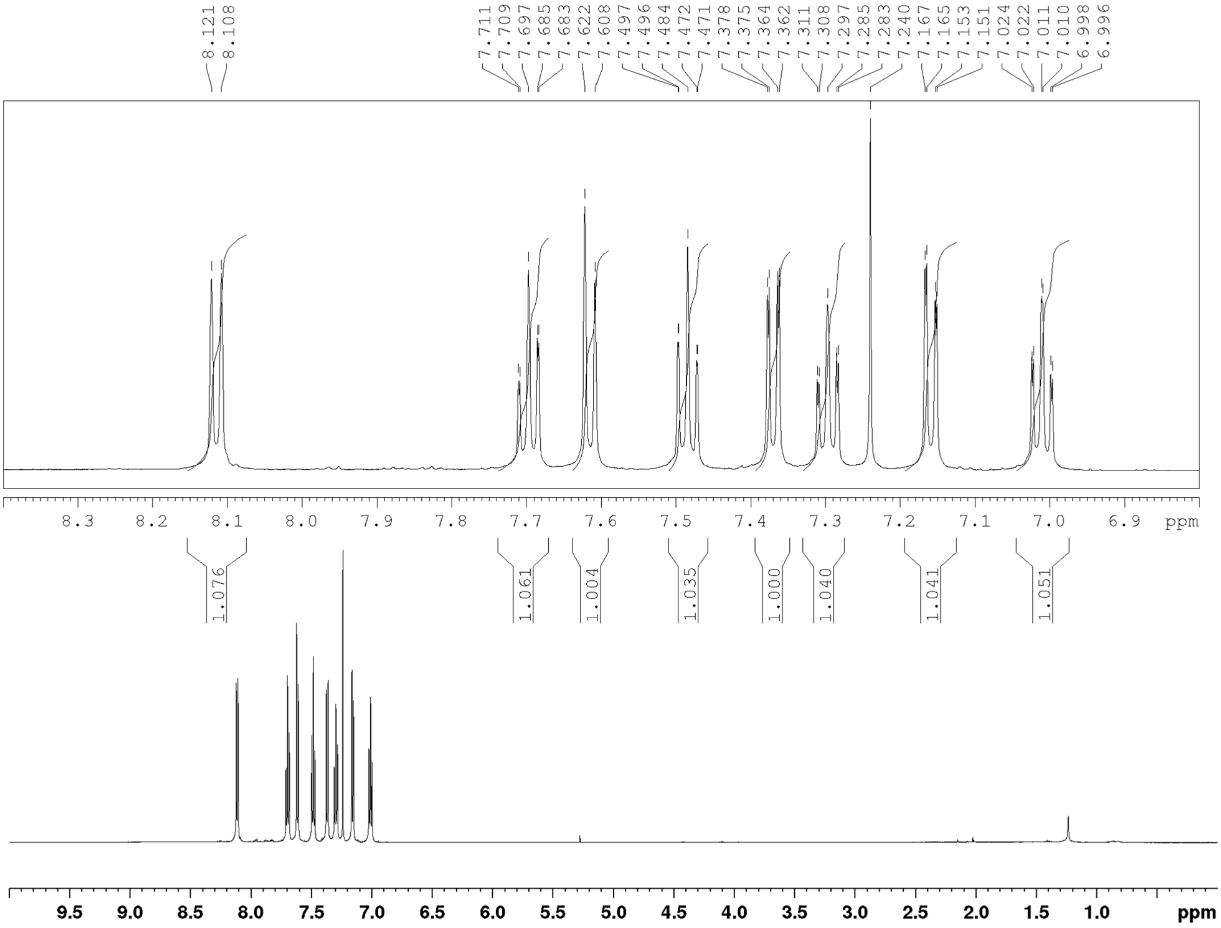
8.121
8.108
7.711
7.709
7.697
7.685
7.683
7.622
7.608
7.497
7.496
7.484
7.472
7.471
7.378
7.375
7.364
7.362
7.311
7.308
7.297
7.285
7.283
7.240
7.167
7.165
7.153
7.151
7.024
7.022
7.011
7.010
6.998
6.996
8.3 8.2 8.1 8.0 7.9 7.8 7.7 7.6 7.5 7.4 7.3 7.2 7.1 7.0 6.9 ppm
1.076
1.061
1.004
1.035
1.000
1.040
1.041
1.051
9.5 9.0 8.5 8.0 7.5 7.0 6.5 6.0 5.5 5.0 4.5 4.0 3.5 3.0 2.5 2.0 1.5 1.0 ppm

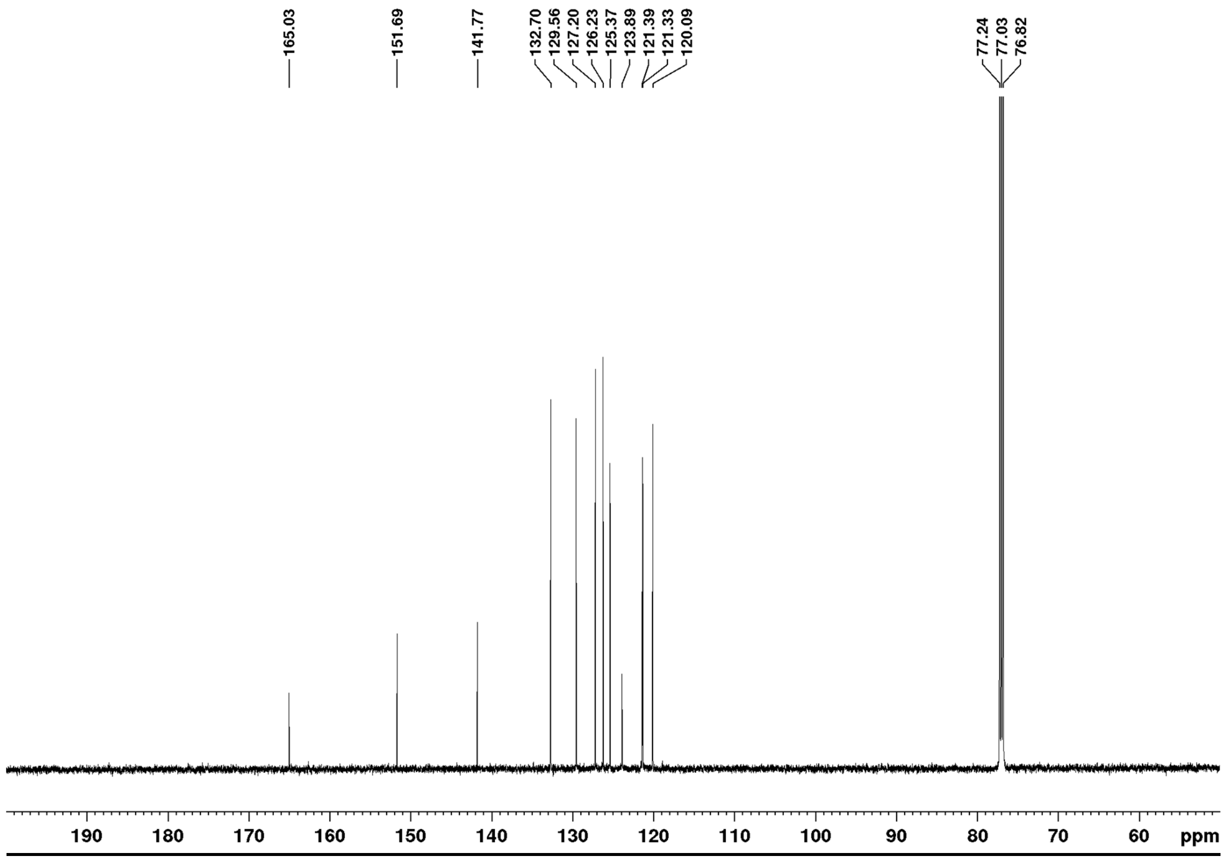
165.03
151.69
141.77
132.70
129.56
127.20
126.23
125.37
123.89
121.39
121.33
120.09
77.24
77.03
76.82
190
180
170
160
150
140
130
120
110
100
90
80
70
60
ppm

(5m)

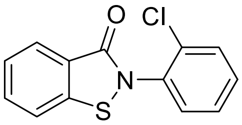
O
Cl
N
S

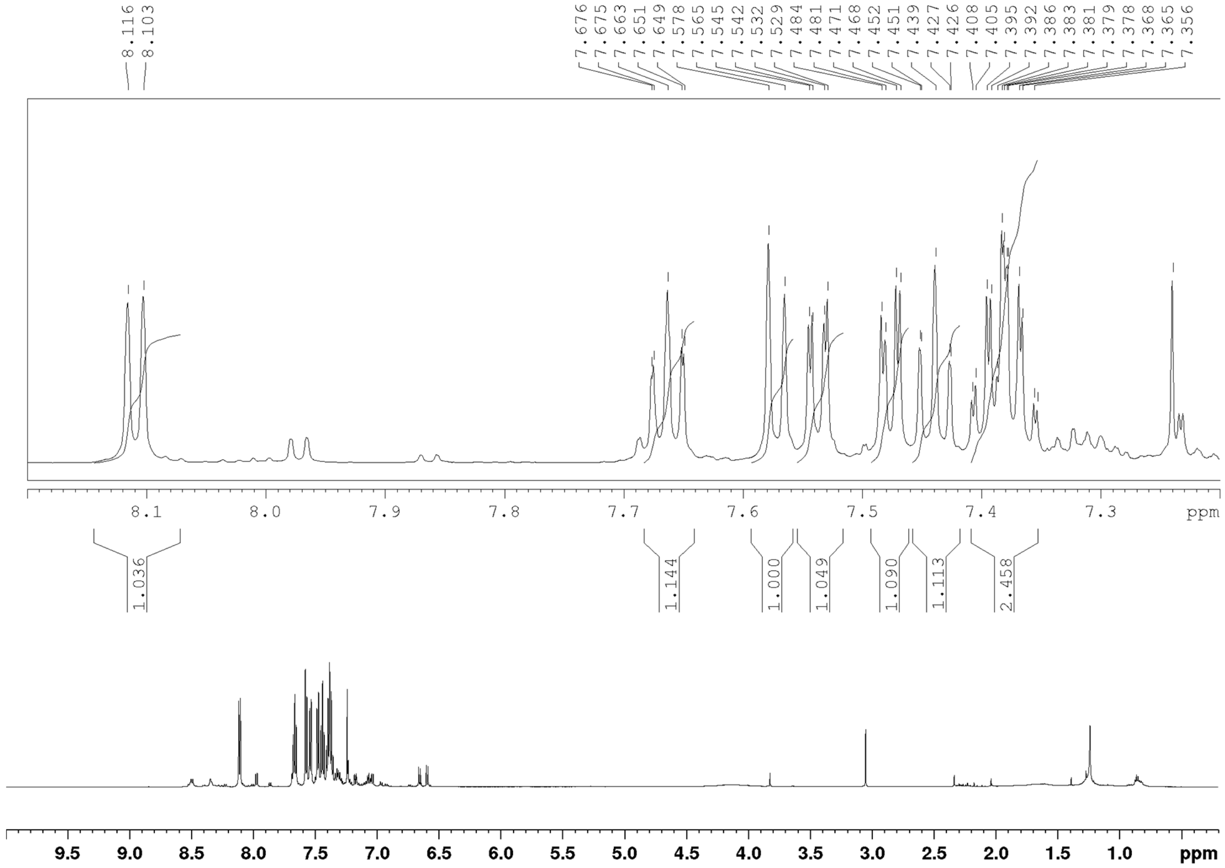
8.116
8.103
7.676
7.675
7.663
7.651
7.649
7.578
7.565
7.545
7.542
7.532
7.529
7.484
7.481
7.471
7.468
7.452
7.451
7.439
7.427
7.426
7.408
7.405
7.395
7.392
7.386
7.383
7.381
7.379
7.378
7.368
7.365
7.356
8.1
8.0
7.9
7.8
7.7
7.6
7.5
7.4
7.3
ppm
1.036
1.144
1.000
1.049
1.090
1.113
2.458
9.5
9.0
8.5
8.0
7.5
7.0
6.5
6.0
5.5
5.0
4.5
4.0
3.5
3.0
2.5
2.0
1.5
1.0
ppm

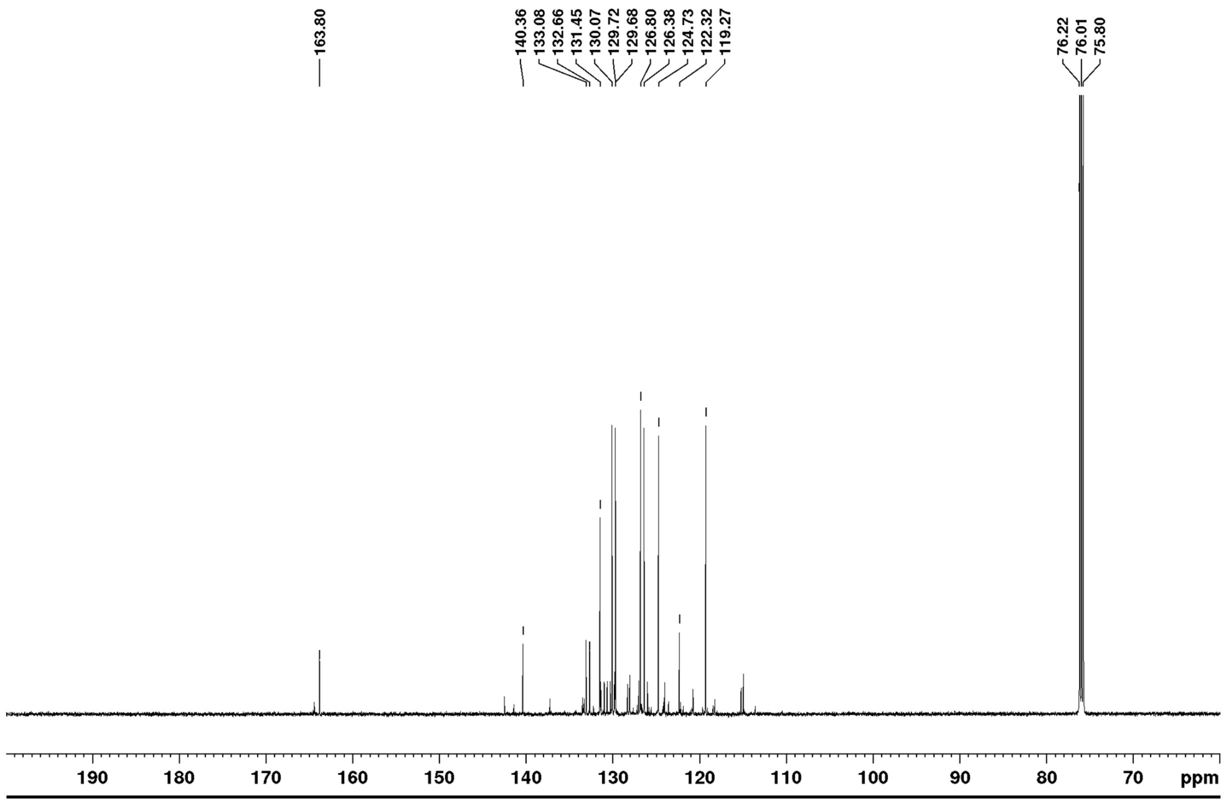
163.80
140.36
133.08
132.66
131.45
130.07
129.72
129.68
126.80
126.38
124.73
122.32
119.27
76.22
76.01
75.80
190
180
170
160
150
140
130
120
110
100
90
80
70
ppm

(5n)

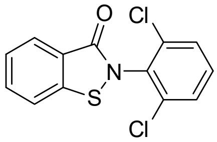
O
Cl
N
S
Cl

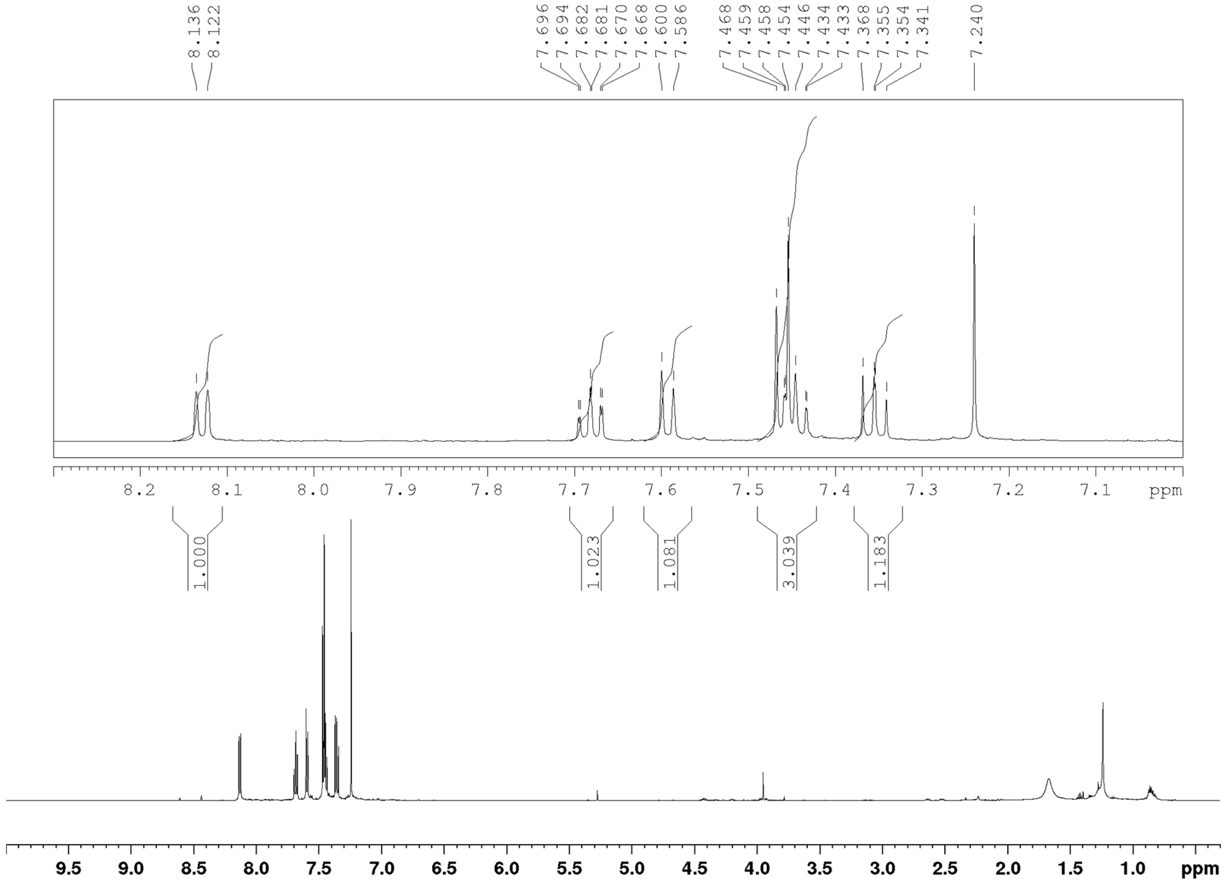
8.136
8.122
7.696
7.694
7.682
7.681
7.670
7.668
7.600
7.586
7.468
7.459
7.458
7.454
7.446
7.434
7.433
7.368
7.355
7.354
7.341
7.240
8.2 8.1 8.0 7.9 7.8 7.7 7.6 7.5 7.4 7.3 7.2 7.1 ppm
1.000
1.023
1.081
3.039
1.183
9.5 9.0 8.5 8.0 7.5 7.0 6.5 6.0 5.5 5.0 4.5 4.0 3.5 3.0 2.5 2.0 1.5 1.0 ppm

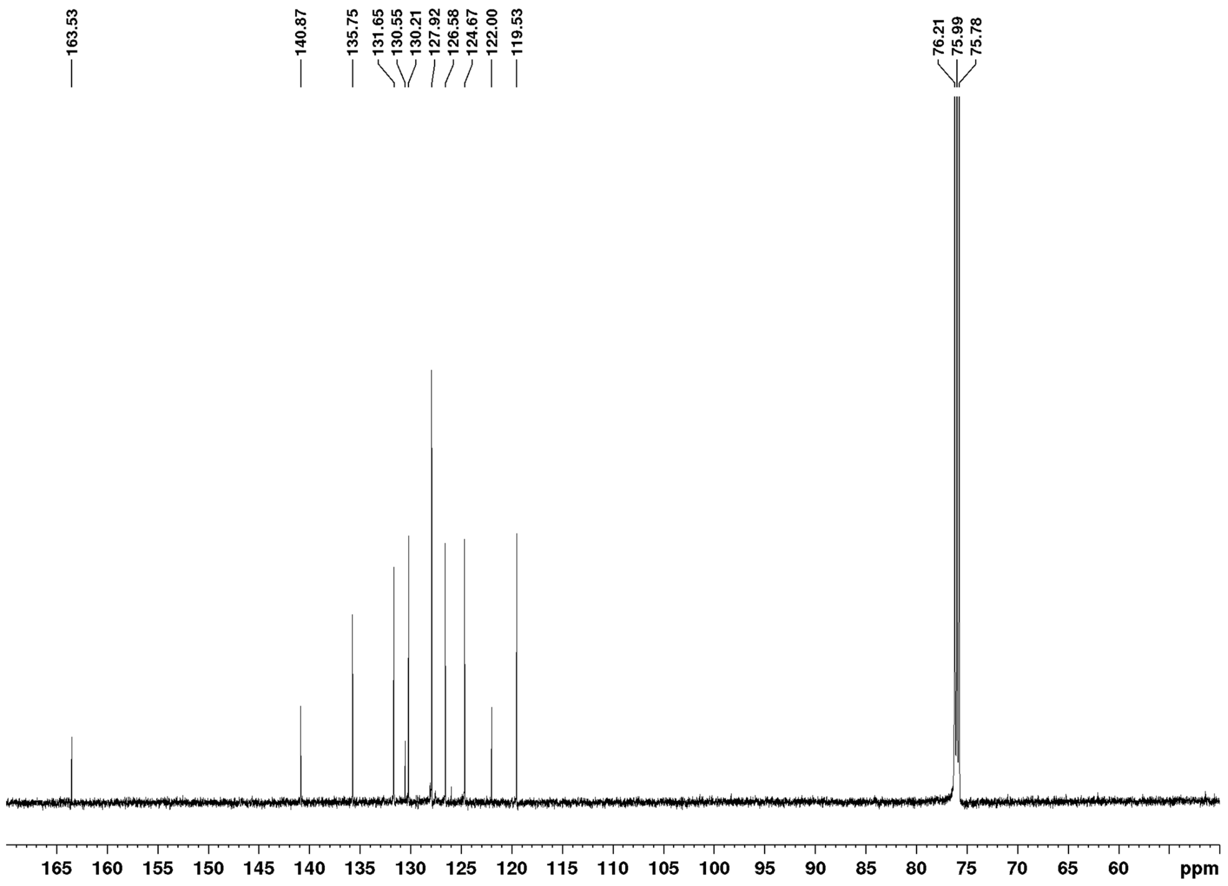
163.53
140.87
135.75
131.65
130.55
130.21
127.92
126.58
124.67
122.00
119.53
76.21
75.99
75.78
165 160 155 150 145 140 135 130 125 120 115 110 105 100 95 90 85 80 75 70 65 60 ppm

(5o)

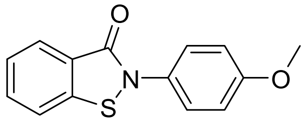

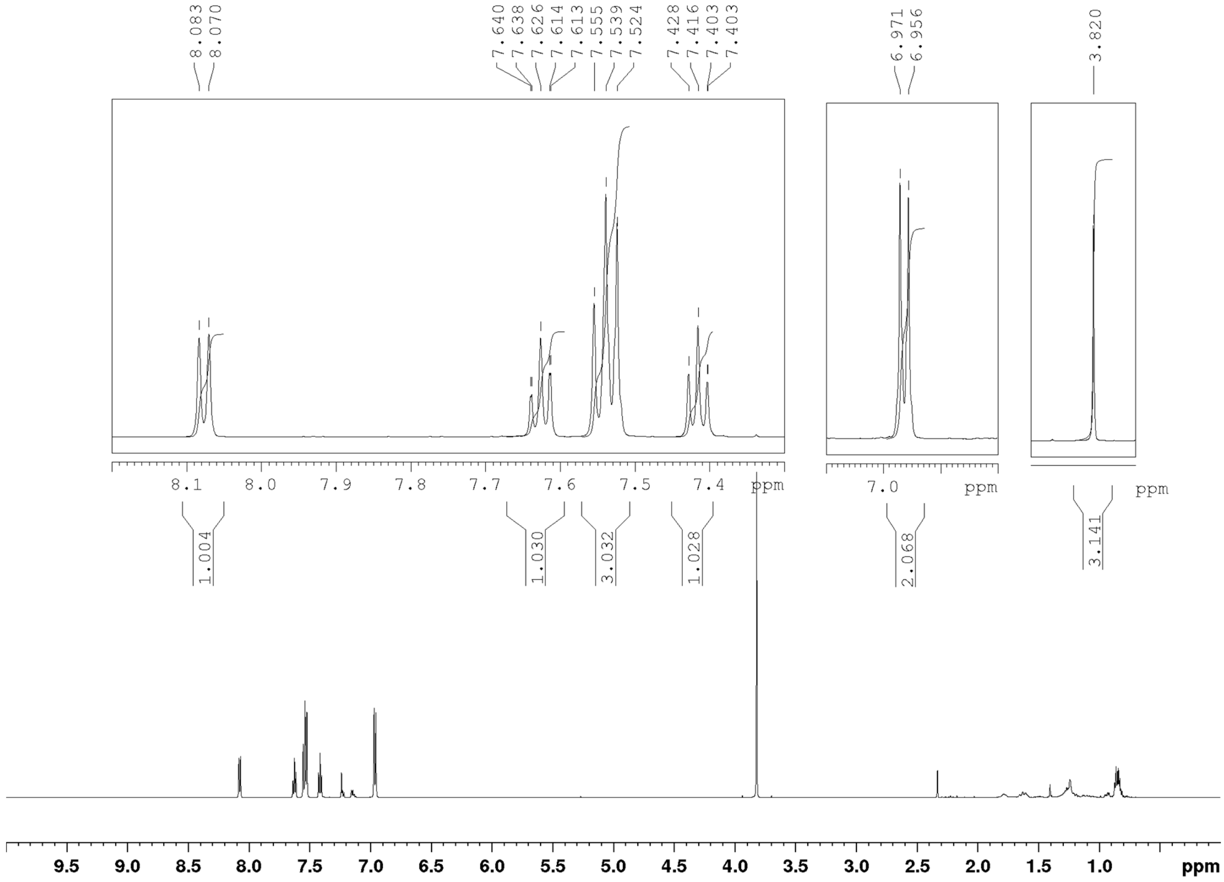
8.083
8.070
7.640
7.638
7.626
7.614
7.613
7.555
7.539
7.524
7.428
7.416
7.403
7.403
6.971
6.956
3.820
8.1
8.0
7.9
7.8
7.7
7.6
7.5
7.4
ppm
7.0
ppm
ppm
1.004
1.030
3.032
1.028
2.068
3.141
9.5
9.0
8.5
8.0
7.5
7.0
6.5
6.0
5.5
5.0
4.5
4.0
3.5
3.0
2.5
2.0
1.5
1.0
ppm

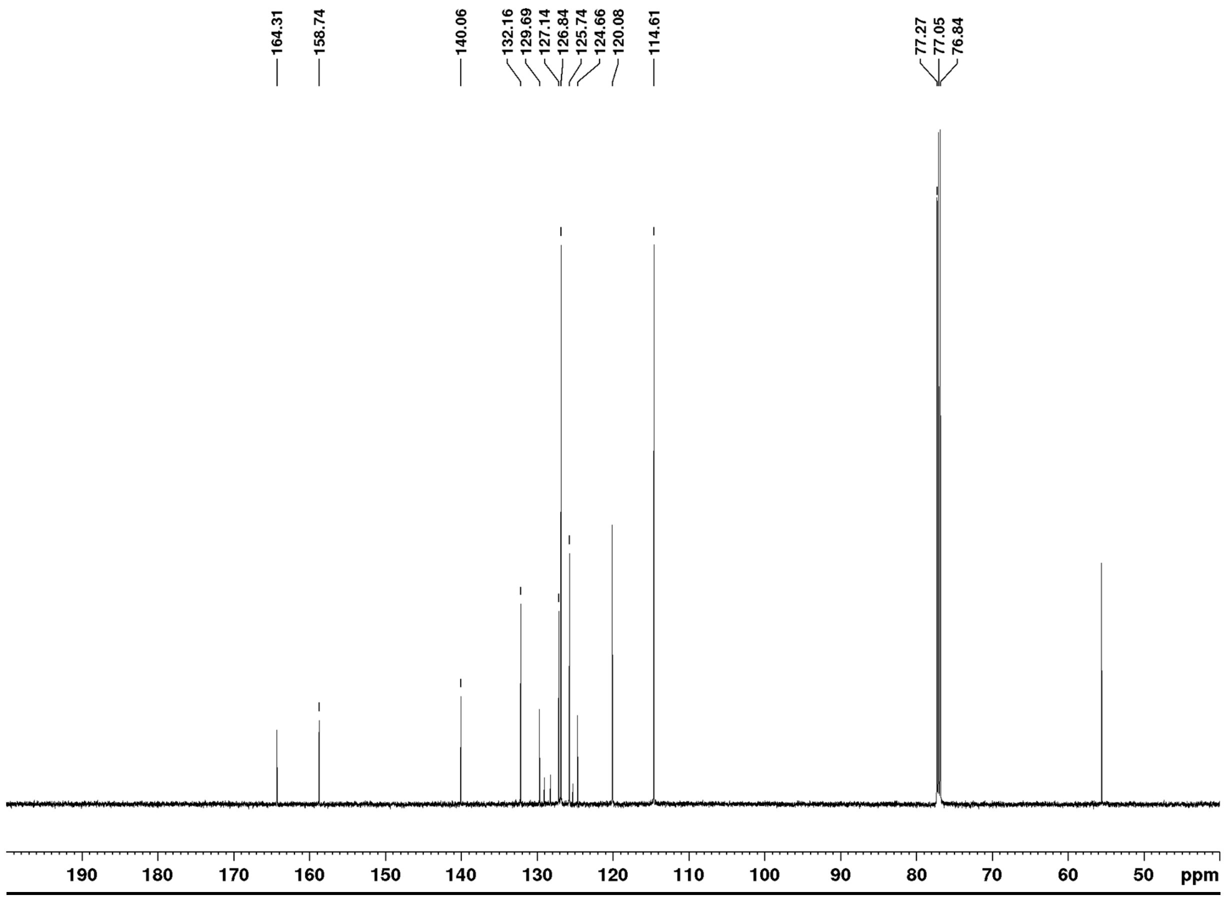
164.31
158.74
140.06
132.16
129.69
127.14
126.84
125.74
124.66
120.08
114.61
77.27
77.05
76.84
190
180
170
160
150
140
130
120
110
100
90
80
70
60
50
ppm
